# Causal effects of nonalcoholic fatty liver disease on cerebral cortical structure: a Mendelian randomization analysis

**DOI:** 10.3389/fendo.2023.1276576

**Published:** 2023-11-01

**Authors:** Zhiliang Mai, Hua Mao

**Affiliations:** ^1^ Department of Digestive Medicine, Zhujiang Hospital, Southern Medical University, Guangzhou, China; ^2^ Department of Anatomy, Guangdong Medical University, Zhanjiang, China

**Keywords:** brain regions, surface area, thickness, mendelian randomization analysis, NAFLD

## Abstract

**Background:**

Previous studies have highlighted changes in the cerebral cortical structure and cognitive function among nonalcoholic fatty liver disease (NAFLD) patients. However, the impact of NAFLD on cerebral cortical structure and specific affected brain regions remains unclear. Therefore, we aimed to explore the potential causal relationship between NAFLD and cerebral cortical structure.

**Methods:**

We conducted a Mendelian randomization (MR) study using genetic predictors of alanine aminotransferase (ALT), NAFLD, and percent liver fat (PLF) and combined them with genome-wide association study (GWAS) summary statistics from the ENIGMA Consortium. Several methods were used to assess the effect of NAFLD on full cortex and specific brain regions, along with sensitivity analyses.

**Results:**

At the global level, PLF nominally decreased SA of full cortex; at the functional level, ALT presented a nominal association with reduced SA of parahippocampal gyrus, TH of pars opercularis, TH of pars orbitalis, and TH of pericalcarine cortex. Besides, NAFLD presented a nominal association with reduced SA of parahippocampal gyrus, TH of pars opercularis, TH of pars triangularis and TH of pericalcarine cortex, but increased TH of entorhinal cortex, lateral orbitofrontal cortex and temporal pole. Furthermore, PLF presented a nominal association with reduced SA of parahippocampal gyrus, TH of pars opercularis, TH of cuneus and lingual gyrus, but increased TH of entorhinal cortex.

**Conclusion:**

NAFLD is suggestively associated with atrophy in specific functional regions of the human brain.

## 1 Introduction

Non-alcoholic fatty liver disease (NAFLD) has emerged as one of the most common chronic liver diseases, affecting 32.4% of the world population (1). It is generally defined as a spectrum of diseases, ranging from nonalcoholic fatty liver, nonalcoholic steatohepatitis (NASH), and liver cirrhosis, which can increase the risk of hepatocellular carcinoma (2). In addition, recent studies indicated that NAFLD is a multi-system disease that can affect various organs and systems, including kidney dysfunction (3), cardiovascular diseases (4), and extrahepatic tumors (5).

Neuropsychiatric diseases are considered as one of the manifestations of NAFLD, such as dementia, depression, and impaired brain health. To be more specific, a system review revealed that patients with NAFLD had an overall 1.44-fold increased risk of cognitive impairment compared with healthy controls (6). Moreover, a study demonstrated that NAFLD constitutes an independent risk factor for anxiety and depression. Besides, previous works have demonstrated close interplays between NAFLD and brain health, including smaller total brain volume, decreased cerebral blood blow and greater arterial stiffness (7–9). These findings suggest there are associations between NAFLD and the whole brain. However, it is also important to identify the link between specific brain region and NAFLD, which may know more about the mechanism of liver-brain axis, and help pave the way to the treatment target of dementia.

The human cerebral cortex, the outer gray matter layer of the brain, plays an important role in cognitive function. Surface area (SA) and thickness (TH) are regarded as important indicators of the human cerebral cortex to study the associations between the brain and the neuropsychiatric diseases (10). Given the uncertainty about the effect of NAFLD on the specific brain regions, further studies that explore the potential impact of NAFLD on the health of specific brain region are warranted.

Mendelian randomization (MR) is an analytic method that uses genetic variants as instruments to estimate the causal effect of risk factors on outcomes (11). MR has become an important method in the recent medical literature because it can overcome the limitations of observational analyses, which are often biased by confounding factors. To date, the use of MR has succeeded in assessing causal relationships in the studies of NAFLD, including several risk factors of NAFLD (12, 13) and relationship between NAFLD and other diseases (14, 15). However, to the best of our knowledge, the causal relation between NAFLD and cerebral cortical structure has not been demonstrated yet.

Hence, the present study used human genetic data within the MR framework to reveal the effect of NAFLD on the SA and TH of full cortex. We also carried out subgroup analyses based on specific brain regions. Considering NAFLD is closely associated with alanine transaminase (ALT) and percent liver fat (PLF), we also selected ALT and PLF as exposures. In the end, three sets of parameters: ALT, NAFLD, and PLF, were used to conduct the MR estimates. Our results shed light on the patterns and mechanisms of brain damage caused by NAFLD and provided new insights into the possible existence of a liver-brain axis.

## 2 Materials and methods

### 2.1 Exposure data

#### 2.1.1 Alanine transaminase

We obtained the summary statistics of ALT from a recent genome-wide association study (GWAS) by Pazoki Raha et al. (16), which included 437,267 individuals of European ancestry. Of note, the ALT levels were log 10 transformed to approximate normal distribution (corresponding to per 10 times of ALT) in the original article.

#### 2.1.2 Non-alcoholic fatty liver disease

Genetic associations with NAFLD were extracted from the largest GWAS meta-analysis to date, which consisted of 8,434 NAFLD cases and 770,180 controls of European ancestry, comprising data from 4 cohorts: Electronic Medical Records and Genomics (eMERGE), UK Biobank, FinnGen and Estonian Biobank (17). In the eMERGE cohort, NAFLD was defined by the use of electronic health record (EHR) codes (ICD-9: 571.5, ICD9: 571.8, ICD-9: 571.9, ICD-10: K75.81, ICD-10: K76.0 and ICD-10: K76.9). In the UK Biobank and Estonian Biobank, NAFLD diagnosis was established from hospital records (ICD-10: K74.0 and K74.2 [hepatic fibrosis], K75.8 [non-alcoholic steatohepatitis], K76.0 [NAFLD] and K76.9 [other specified diseases of the liver]). In the FinnGen Consortium, NAFLD was defined by EHR code K76.0.

#### 2.1.3 Percent liver fat

Genetic associations with PLF were extracted from a GWAS from a cohort (18) that consisted of 32,858 European participants from the UK Biobank. The cohort used deep learning to process over 38,000 abdominal MRI scans to quantify volume, fat, and iron in seven organs and tissues, including the liver. The GWAS of PLF adjusted for several covariates, including age at imaging visit, age squared, sex, imaging center, scan date, scan time, genotyping batch, and genetic relatedness.

### 2.2 Outcome data

We obtained the GWAS data for SA and TH from the ENIGMA Consortium (19). The ENIGMA Consortium conducted a genome-wide association meta-analysis study on cortical structures, which included the SA and TH of the full cortex, as well as SA and TH for thirty-four brain cortical regions with known functional specializations. The thirty-four brain regions were defined using the Desikan-Killiany cortical atlas, and established coarse partitions of the cortex. The SA and TH were measured using MRI in 51,665 individuals from 60 cohorts around the world, with approximately 94% of European descent. Both SA and TH of brain regions were weighted by the entire brain, indicating the SA and TH of specific regions across the SA and TH of the entire brain. These data can be accessed at https://enigma.ini.usc.edu/research/download-enigma-gwas-results/. All GWASs data used in the study are shown in **Table S1**.

### 2.3 Instrumental variable selection

**Figure 1:**
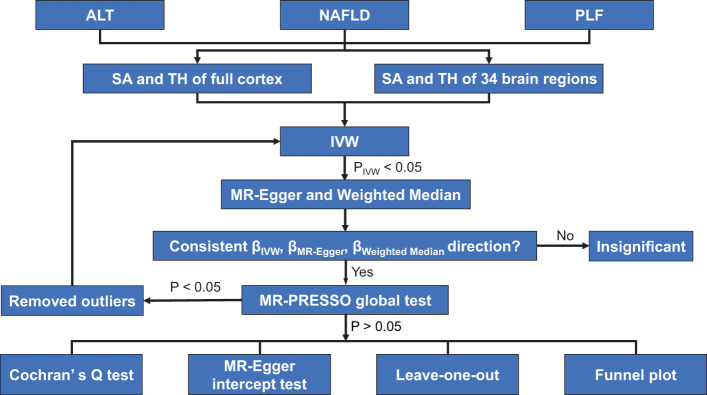
Study flame chart of the Mendelian randomization study revealing the causal relationship between alanine transaminase, non-alcoholic fatty liver disease, and percent liver fat and the cerebral cortical structure. ALT, alanine transaminase*;* IVW, inverse-variance weighted; MR-PRESSO, Mendelian randomization-pleiotropy residual sum and outlier; NAFLD, non-alcoholic fatty liver disease; PLF, percent liver fat; SA, surface area; TH, thickness.

To identify the causal relationship between NAFLD and the cerebral cortical structure, we used three sets of genetic instruments, including: i) index Single Nucleotide Polymorphisms (SNP) representing ALT, ii) index SNPs representing NAFLD, and iii) index SNPs representing PLF. Genetic instruments were selected via the following criteria: i) a GWAS-correlated P-value of 5 × 10^-8^, ii) the minor allele frequency (MAF) threshold of the variants of interest was 0.01, iii) a linkage disequilibrium (LD) r^2^ of < 0.001, and < 10 MB from the index variant, iv) an F statistic of 10 was regarded as sufficiently robust to counteract weak instrument bias. Finally, when no SNP in the outcome dataset met this criterion, proxy SNPs with LD set at r^2^ > 0.8 were used. The study flow chart is presented in **Figure 1**.

### 2.4 Ethics

This study used publicly available data from participant studies that were approved by an ethical standards committee with respect to human experimentation. No separate ethical approval was required in this study.

### 2.5 MR analysis

Three different methods of MR [inverse-variance weighted (IVW), MR Egger, and weighted median] were performed to address variant heterogeneity and the pleiotropy effect. IVW was used as the main analysis, because it is reported to be slightly more powerful than other methods under certain conditions (20). However, IVW assumes that all genetic variants are valid instruments (21), which may not be true in practice. Therefore, MR-Egger and weighted median were used as complements to improve the IVW estimates as they could provide more robust estimates in a broader set of scenarios. MR-Egger method allows all genetic variants to have pleiotropic effect but requires that the pleiotropic effects be independent of the variant-exposure association (22). Weighted median allows for the use of invalid instruments when less than half of the instruments used in the MR analysis are valid (22). We only performed MR-Egger and weighted median when P_IVW_ < 0.05. When all methods had consistent β directions, the effect estimates were considered significant (23). For significant estimates, we further assessed horizontal pleiotropy using the MR-PRESSO global test (24, 25). If the SNP was identified by MR-PRESSO outlier test as outliers, it would be removed and then the MR analysis was re-performed. Additionally, as the MR estimate may be biased in the present of invalid instruments, several sensitivity analyses were performed. MR-Egger regression test was used to obtain the intercept, which was an indicator for directional pleiotropy (P < 0.05 was considered as the presence of directional pleiotropy) (26). Funnel plots were used to assess the probable pleiotropy and heterogeneity. The Cochran’s Q test was also used to evaluate heterogeneity (27).

Additionally, we established a multiple testing significance threshold at different outcome (full cortex, specific brain regions), defined as P < 0.05/(3×n) (where n is the number of outcomes). Therefore, a P value less than 8.3 × 10^−3^ (0.05/6) was considered statistically significant in the estimation of SA and TH of full cortex, while a P value less than 2.5 × 10^−4^ (0.05/204) was considered statistically significant in the estimation of SA and TH of certain brain region. A P value less than 0.05 was considered nominally significant evidence for a potential causal association (23, 28, 29). All analyses were performed using the package TwoSampleMR (30) (version 0.5.6) and package MRPRESSO (25) (version 1.0) in R (version 4.1.3).

## 3 Results

In total, 10 index SNPs were selected to genetically predict ALT, 10 index SNPs were used to genetically predict PLF and 4 SNPs predict NAFLD. F statistics for these genetic instruments were all larger than the normally selected value of 10, ranging from 10.6 to 23,115.9, indicating no evidence of no weak instruments (31). SNP rs429358 was overlapped in PLF and NAFLD. SNP rs58542926 was overlapped in ALT and PLF. There was no overlapping between ALT and NAFLD. All the details about the SNPs were shown in the **Table S2**.

**Figure 2:**
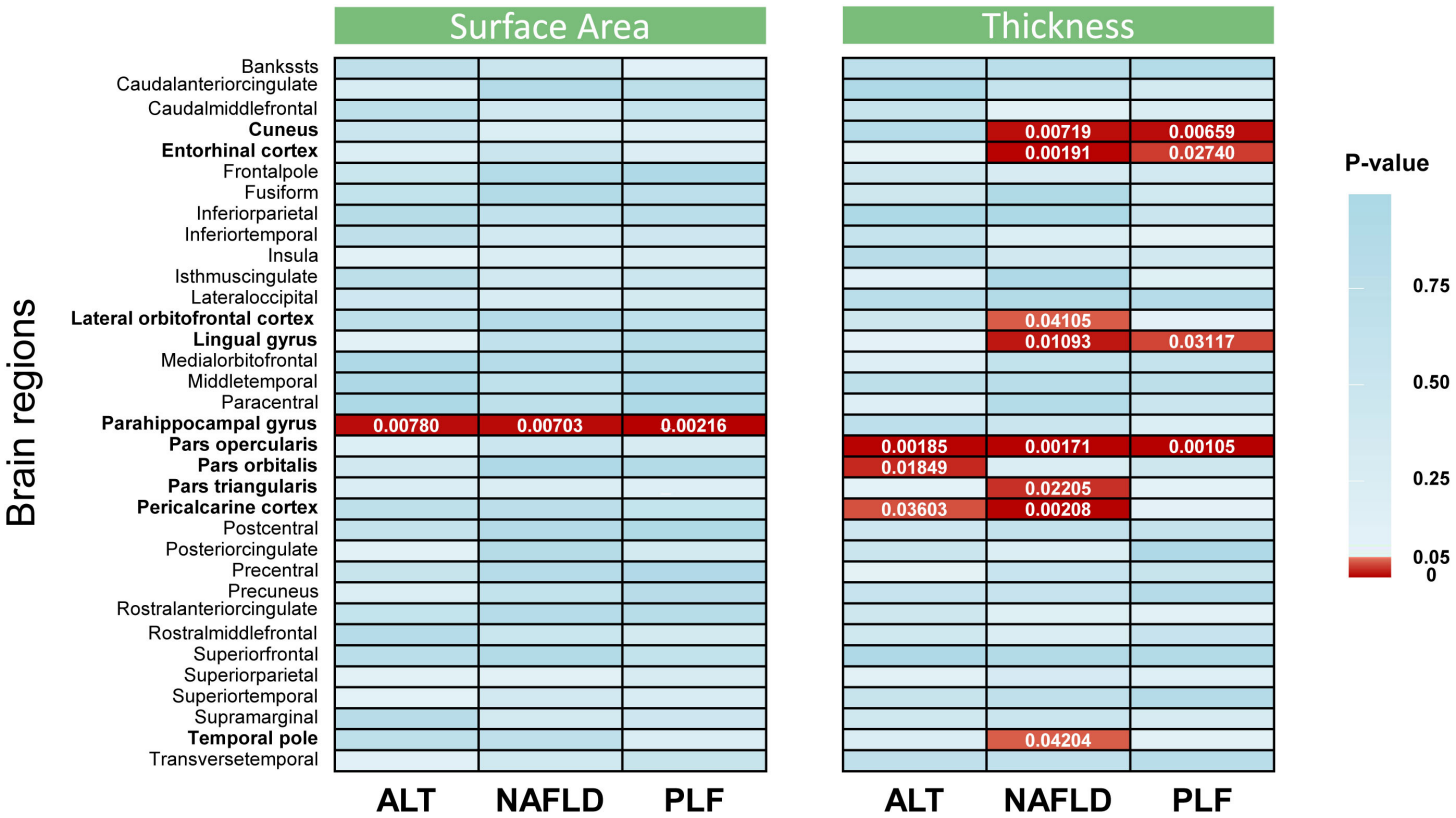
Inverse-variance weighted estimates from alanine transaminase, non-alcoholic fatty liver disease, and percent liver fat on cerebral cortical structure. The color of each block represented the p value of each analysis: red blocks indicated p < 0.05 and blue blocks indicated p ≥ 0.05. A p value < 2.5 × 10^−4^ was considered statistically significant, while a p value < 0.05 was considered nominally significant. The brain regions whose p values were less than 0.05 are highlighted in bold in the left of the figure. ALT, alanine transaminase; NAFLD, non-alcoholic fatty liver disease; PLF, percent liver fat.

**Figure 3:**
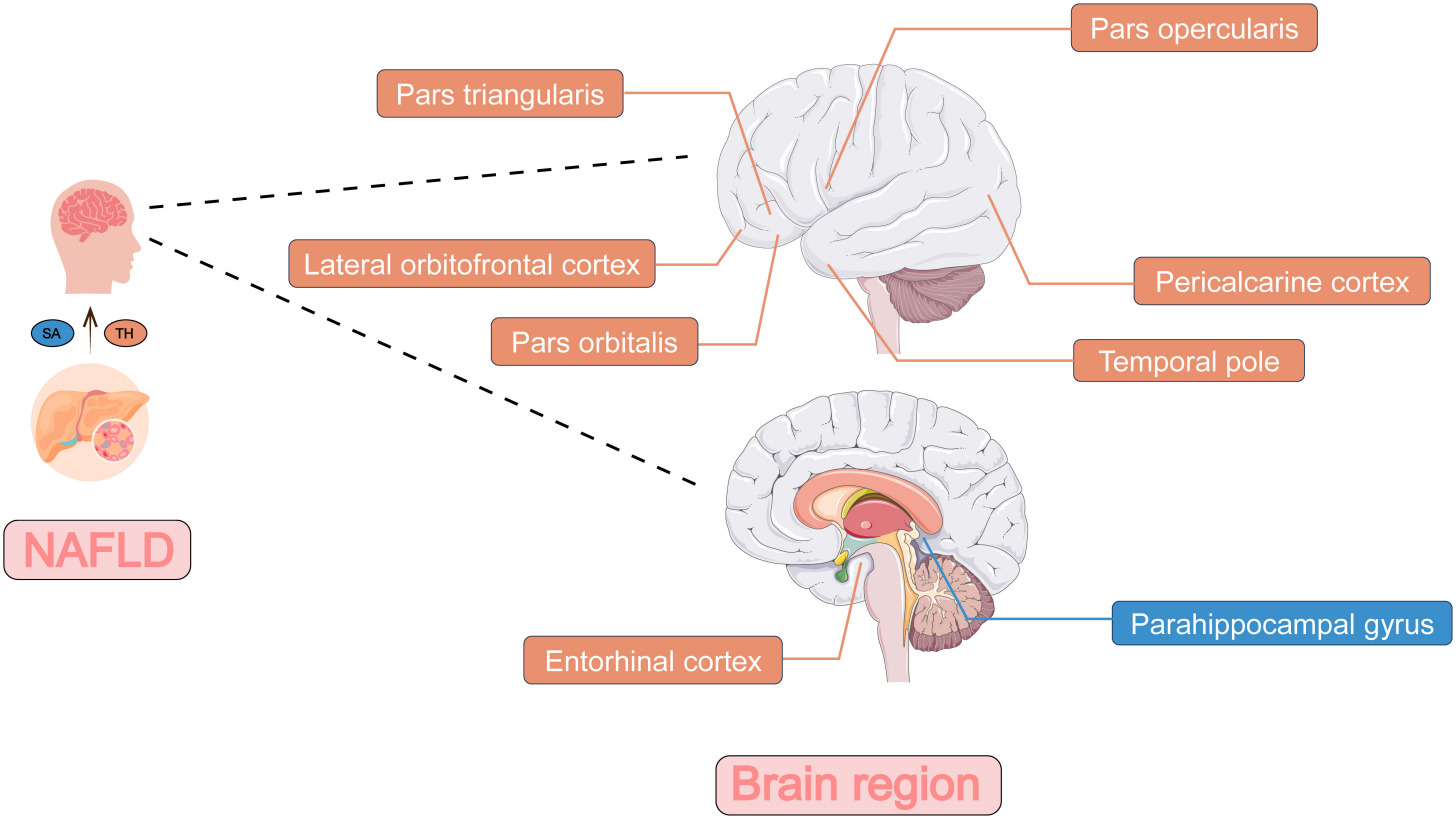
The two-sample Mendelian randomization framework showed that NAFLD potentially influenced cerebral cortical structure. The influence on the surface area of brain regions was shown in blue and the influence on the thickness of brain regions was shown in orange. NAFLD, non-alcoholic fatty liver disease; SA, surface area; TH, thickness.

MR analysis was performed to evaluate the causal relationships of NAFLD with SA and TH of brain region and full cortex (**Figure 1**). **Table 1** showed the NAFLD’s causal effect on the full cortex. All the results about the main analysis were presented in **Table S3** and **Figure 2**. **Table 2** and **Figure 3** showed the nominally significant brain regions affected by NAFLD.

**Table 1:** Mendelian randomization estimates from alanine transaminase, non-alcoholic fatty liver disease and percent liver fat on genetically predicted full cortex.

Exposures Outcomes	Method	β (95%CI)	SE	P value
ALT				
Surface area of full cortex	IVW	-6700.0683 (-15685.3599, 2285.2232)	4584.3320	0.14387
Thickness of full cortex	IVW	-0.0338 (-0.0921, 0.0244)	0.0297	0.25534
NAFLD				
Surface area of full cortex	IVW	-880.5200 (-1775.4473, 14.4072)	456.5955	0.05380
Thickness of full cortex	IVW	-0.0019 (-0.0076, 0.0038)	0.0029	0.51819
PLF				
Surface area of full cortex	IVW	-900.7396 (-1625.1751, -176.3041)	369.6100	0.01481
Thickness of full cortex	IVW	-0.0047 (-0.0105, 0.0011)	0.0030	0.11007

ALT, alanine transaminase; IVW, inverse-variance weighted; NAFLD, non-alcoholic fatty liver disease; PLF, percent liver fat; SE, Standard error.

**Table 2:** Mendelian randomization estimates from alanine transaminase, non-alcoholic fatty liver disease and percent liver fat on genetically predicted specific brain regions.

Exposures Outcomes	Method	β (95%CI)	SE	P value
ALT				
Surface area of parahippocampal gyrus	IVW	-60.4594 (-104.9948, -15.9239)	22.72216	0.00780
Thickness of pars opercularis	IVW	-0.0861 (-0.1402, -0.0319)	0.02764	0.00185
Thickness of pars orbitalis	IVW	-0.1023 (-0.1874, -0.0172)	0.04343	0.01849
Thickness of pericalcarine cortex	IVW	-0.0913 (-0.1767, -0.006)	0.04356	0.03603
NAFLD				
Surface area of parahippocampal gyrus	IVW	-5.3315 (-9.2083, -1.4547)	1.97796	0.00703
Thickness of cuneus	IVW	-0.0075 (-0.0130, -0.0020)	0.00280	0.00719
Thickness of entorhinal cortex	IVW	0.0251 (0.0093,0.0410)	0.00810	0.00191
Thickness of lateral orbitofrontal cortex	IVW	0.0062 (0.0003,0.0121)	0.00303	0.04105
Thickness of lingual gyrus	IVW	-0.0063 (-0.0112, -0.0015)	0.00249	0.01093
Thickness of pars opercularis	IVW	-0.0072 (-0.0117, -0.0027)	0.00230	0.00171
Thickness of pars triangularis	IVW	-0.0058 (-0.0109, -0.0008)	0.00255	0.02205
Thickness of pericalcarine cortex	IVW	-0.0086 (-0.0141, -0.0031)	0.00280	0.00208
Thickness of temporal pole	IVW	0.0143 (0.0005, 0.0281)	0.00703	0.04204
PLF				
Surface area of parahippocampal gyrus	IVW	-6.0644 (-9.9393, -2.1895)	1.97698	0.00216
Thickness of cuneus	IVW	-0.0077 (-0.0132, -0.0021)	0.00282	0.00659
Thickness of entorhinal cortex	IVW	0.0246 (0.0027, 0.0465)	0.01115	0.02740
Thickness of lingual gyrus	IVW	-0.0063 (-0.0119, -0.0006)	0.00290	0.03117
Thickness of pars opercularis	IVW	-0.0077 (-0.0123, -0.0031)	0.00235	0.00105

ALT, alanine transaminase; IVW, inverse-variance weighted; NAFLD, non-alcoholic fatty liver disease; PLF, percent liver fat; SE, Standard error.

As is shown in the **Table 1**, PLF was found to decrease SA of full cortex (*β* = -900.7396 mm^2^, 95% CI: -1625.1751 mm^2^ to -176.3041 mm^2^, *p* = 0.01481) but had no causal relationship with TH (*β* = -0.0047 mm, 95% CI: -0.0105 mm to 0.0011 mm, *p* = 0.11007). Heterogeneity was not observed by Cochran’s Q test (*p* = 0.93, **Table 3**). The P value for MR-Egger intercept is 0.32, indicating there is no pleiotropy (**Table 3**). ALT had no causal relationship with the SA and TH of full cortex (*β*_SA_ = -6700.0683 mm^2^, 95% CI: -15685.3599 mm^2^ to 2285.2232 mm^2^, *p*_SA_ = 0.14387; *β*_TH_ = -0.0338 mm, 95% CI: -0.0921 mm to 0.0244 mm, *p*_TH_ = 0.25534). Genetic predicted NAFLD had no causal relationship with the SA and TH of full cortex (*β*_SA_ = -880.5200 mm^2^, 95% CI: -1775.4473 mm^2^ to 14.4072 mm^2^, *p*_SA_ = 0.05380; *β*_TH_ = -0.0019 mm, 95% CI: -0.0076 mm to 0.0038 mm, *p*_TH_ = 0.51819).

**Table 3:** Heterogeneity and pleiotropy tests of the significant and nominally significant Mendelian randomization estimates.

Exposures Outcomes	Cochrane’s Q test		MR−Egger intercept test	
	Q-value	P_Q_	Intercept	P_Intercept_
ALT				
Surface area of parahippocampal gyrus	1.88701	0.93	0.38531	0.64
Thickness of pars opercularis	6.1745	0.4	0.00079	0.45
Thickness of pars orbitalis	1.67879	0.95	-0.00064	0.69
Thickness of pericalcarine cortex	11.38044	0.08	0.00295	0.04
NAFLD				
Surface area of parahippocampal gyrus	0.65043	0.72	-1.02464	0.65
Thickness of entorhinal cortex	0.48841	0.78	-0.00475	0.61
Thickness of lateral orbitofrontal cortex	0.1001	0.95	0.00077	0.81
Thickness of pars opercularis	0.18859	0.91	-0.00068	0.79
Thickness of pars triangularis	0.32784	0.85	-0.00088	0.76
Thickness of pericalcarine cortex	0.04032	0.98	0.00039	0.9
Thickness of temporal pole	0.76527	0.68	0.00015	0.98
PLF				
Surface area of full cortex	3.09366	0.93	76.57317	0.32
Surface area of parahippocampal gyrus	2.17483	0.98	-0.41264	0.32
Thickness of cuneus	6.49498	0.59	0.0002	0.73
Thickness of entorhinal cortex	15.07717	0.06	0.00026	0.91
Thickness of lingual gyrus	10.98328	0.2	0.00005	0.93
Thickness of pars opercularis	3.93683	0.86	0.00008	0.86

ALT, alanine transaminase; NAFLD, non-alcoholic fatty liver disease; PLF, percent liver fat.

### 3.1 Causal estimates of genetically predicted ALT on the brain regions

The genetically predicted ALT was nominally associated with reduced SA in the parahippocampal gyrus (*β* = -60.4594 mm^2^, 95% CI: -104.9948 mm^2^ to -15.9239 mm^2^, *p* = 0.0078). A similar result was obtained when analyzing index SNPs predicted ALT and TH of several brain regions, including pars opercularis (*β* = -0.0861 mm, 95% CI: -0.1402 mm to -0.0319 mm, *p* = 0.00185), pars orbitalis (*β* = -0.1023 mm, 95% CI: -0.1874 mm to -0.0172 mm, *p* = 0.01849) and pericalcarine cortex (*β* = -0.0913 mm, 95% CI: -0.1767 mm to -0.006 mm, *p* = 0.03603).

### 3.2 Causal estimates of genetically predicted NAFLD on the brain regions

The genetically predicted NAFLD was nominally associated with reduced SA in the parahippocampal gyrus (*β* = -5.3315 mm^2^, 95% CI: -9.2083 mm^2^ to -1.4547 mm^2^, *p* = 0.00703). NAFLD was also found to be nominally associated with reduced TH of cuneus (*β* = -0.0075 mm, 95% CI: -0.0130 mm to -0.0020 mm, *p* = 0.00719), lingual gyrus (*β* = -0.0063 mm, 95% CI: -0.0112 mm to -0.0015 mm, *p* = 0.01093), pars opercularis (*β* = -0.0072 mm, 95% CI: -0.0117 mm to -0.0027 mm, *p* = 0.00171), pars triangularis (*β* = -0.0058 mm, 95% CI: -0.0109 mm to -0.0008 mm, *p* = 0.02205), and pericalcarine cortex (*β* = -0.0086 mm, 95% CI: -0.0141 mm to -0.0031 mm, *p* = 0.00208). However, there was nominally significant evidence that the NAFLD was associated with increased TH of the entorhinal cortex (*β* = 0.0251 mm, 95% CI: 0.0093 mm to 0.0410 mm, *p* = 0.00191), lateral orbitofrontal cortex (*β* = 0.0062 mm, 95% CI: 0.0003 mm to 0.0121 mm, *p* = 0.04105) and temporal pole (*β* = 0.0143 mm, 95% CI: 0.0005 mm to 0.0281 mm, *p* = 0.04204).

### 3.3 Causal estimates of genetically predicted PLF on the brain regions

The PLF was nominally associated with reduced SA of the parahippocampal gyrus (*β* = -6.0644 mm^2^, 95% CI: -9.9393 mm^2^ to -2.1895 mm^2^, *p* = 0.00216). In addition, genetic predisposition to PLF was nominally associated with decreased TH in several regions, including the cuneus (*β* = -0.0077 mm, 95% CI: -0.0132 mm to -0.0021 mm, *p* = 0.00659), lingual gyrus (*β* = -0.0063 mm, 95% CI: -0.0119 mm to -0.0006 mm, *p* = 0.03117), and pars opercularis (*β* = -0.0077 mm, 95% CI: -0.0123 mm to -0.0031 mm, *p* = 0.00105). However, a positive association was obtained when analyzing index SNPs predicted PLF and TH of entorhinal cortex (*β* = 0.0246 mm, 95% CI: 0.0027 mm to 0.0465 mm, *p* = 0.02740).

### 3.4 Sensitivity analysis

For both significant and nominally significant estimates, we next performed MR-Egger and weighted median analyses. All of these results were directionally consistent with the IVW analyses except for estimates of NAFLD on the TH of cuneus and lingual gyrus (**Table S4**), which were considered as insignificant. For the remaining significant and nominally significant estimates, we performed MR-PRESSO global tests, but no horizontal pleiotropy was detected (**Table S5**). Cochran’s Q test, MR-Egger intercept test, leave-one-out analyses, and funnel plot were also performed. **Table 3** showed that no heterogeneity was detected (all *p*_Q_ > 0.05). Besides, all P-values of MR Egger intercept tests were > 0.05. Scatter plots, leave-one-out analyses and funnel plots were shown in **Supplementarys Figure S1**–**S9**. The estimates were not biased by single SNP, indicating that estimates were not violated.

## 4 Discussion

To the best of our knowledge, our study is the first to determine the causal relationship between NAFLD and the cerebral cortical structure. Our results showed that ALT, PLF, and NAFLD could affect the cerebral cortical structure, and supported the findings of earlier observational studies indicating the pathophysiologic interactions between NAFLD and brain functions, thereby highlighting the existence of the liver-brain axis.

At the global level, we found that genetically predicted PLF was nominally associated with decreased SA of full cortex. To the best of our knowledge, a limited number of studies have published the evaluation of the association between PLF and full cortex. A previous study (8) showed that higher liver fat was associated with decreased total-cerebral blood flow and gray matter- cerebral blood flow, which could be an explanation for our findings. Also, the study revealed that NAFLD was linked with lower total brain volume. However, our findings showed that SNPs predicted NAFLD have no relationship with the SA and TH of full cortex. This could be because only 4 SNPs were used for MR analysis. At the brain region level analysis, the suggestive relationships were mostly about TH of brain regions. This suggested that measuring the TH of brain regions can be a measure to evaluate the extent of damage caused by NAFLD to the brain. Besides, most of the differences in cortical structure observed in intelligence, cognitive function and neuropsychiatric diseases have been reported for TH (32–34), perhaps suggesting that NAFLD causes neuropsychiatric diseases by mediating the destruction of the TH of specific brain regions.

The present study provided evidence that ALT, NAFLD and PLF were all nominally associated with decreased SA of parahippocampal gyrus. The parahippocampal gyrus is an essential site that coordinates with hippocampus (35) to be responsible for memory encoding, storage and retrieval. It has been proved to be vital in the mechanism of several brain diseases and psychiatric condition, such as Posttraumatic stress disorder (36), Alzheimer’s disease (37) and schizophrenia (38). Besides, some studies indicated that liver diseases could have impact on parahippocampal gyrus. Jiang et al. (39) showed that people with advanced liver fibrosis had worse cognitive functioning and decreased grey matter in the hippocampus and parahippocampal gyrus. Chen et al. (40) found that patients with cirrhosis tended to damage parahippocampal gyrus and other gray matter regions, and decrease brain microstructural complexity, which may contribute to the cognitive impairment. The underlying mechanism of alterations of parahippocampal gyrus in patients with liver diseases warrants further investigations. Whether NAFLD will lead to changes of parahippocampal gyrus and thus lead to neuropsychiatric disorders could also be expected in the future studies.

Besides, our study found that the TH of pars opercularis is nominally influenced by ALT, NAFLD and PLF. The nominally causal effect of ALT on pars orbitalis and NAFLD on the TH of pars triangularis were also observed. These parts make up the inferior frontal gyrus, which is a key region in language processing and speech production, along with various cognitive functions, such as motor inhibition (41), response inhibition (42), and social cognitive processes (43). Our findings were consistent with previous studies. Chen et al. (44) suggested there is aberrant spontaneous activity of inferior frontal gyrus in the patients with low-grade hepatic encephalopathy. Yang et al. (45) also found a decreased functional connectivity between right dorsolateral prefrontal cortex and inferior frontal gyrus in the patients with cirrhosis. These studies illustrate the connection between inferior frontal gyrus and liver. However, whether NAFLD will lead to these functional changes or neuropsychiatric disorders mediating the alteration of TH of the three parts could also be expected in the future studies.

NAFLD may have an influence on the morbidity of complications in patients with diabetes, involving diabetic retinopathy (46, 47), which indicated the relationship between the liver and the eyes. Our study also indicates a suggestively significant association between ALT and TH of pericalcarine, as well as association between PLF and TH of cuneus. The pericalcarine, the primary visual cortex, processes the visual signals. Also, the pericalcarine can activate the cuneus, which responds to the visual stimuli (48). Considering the NAFLD may have an influence on the pericalcarine and cuneus, the connection between liver and eyes could probably be explained.

Several mechanisms that NAFLD affects brain health are considered and constantly evolving. (1) liver fat may activate microglial cells in the brain by inducing inflammation, and thus resulting in elevated expression of inflammatory cytokines (49); (2) similar with obesity, patients with NAFLD may also increase brain insulin resistance, thereby causing oxidative stress, excessive free fatty acids, and brain mitochondrial disorders (50); (3) impaired liver function can cause insufficient detoxification and allows neurotoxins to enter the cerebral circulation, which can increase permeability of blood–brain barrier and neuroinflammation (51, 52).

Notably, some of our estimates deviated from logical expectation. NAFLD should lead to a smaller TH of the brain regions. However, in our study, genetically predicted NAFLD leads to increased TH of lateral orbitofrontal cortex, temporal pole and entorhinal cortex. Similarly, PLF correlated with larger TH of entorhinal cortex. The possible explanation may be a compensatory hypertrophy or encephaledema. Further studies are needed to investigate the underlying mechanism.

The primary strength of our study is a comprehensive MR study, which can overcome the shortcomings of observational studies. Our study assessed the associations between NAFLD and specific brain regions, and may pave the way to understand the mechanisms that link NAFLD to dementia and other neuropsychiatric diseases. This is essential to achieving more optimized surveillance and providing treatments for patients with NAFLD. However, this study has several limitations. First, the groups in our study were all European, and the conclusions in other populations should be interpreted with caution. Second, the present study did not investigate severity of the changes of brain region. Third, the underlying mechanisms of the change of brain regions warrant further investigation. Future studies should investigate the mechanism underlying the association between NAFLD and neuropsychiatric diseases to explore novel treatments for neuropsychiatric disorders in patients with NAFLD.

## 5 Conclusion

This is the first comprehensive MR analysis that reveals associations between NAFLD and the cerebral cortical structure. Our estimates illustrate that NAFLD suggestively decreases specific functional regions of the human brain. For patients with NAFLD, a brain MRI could potentially be used for early diagnosis of neuropsychiatric disorders. The mechanisms of the association between NAFLD and brain function alterations should be studied further.

## Data availability statement

All GWAS data are available and can be freely downloaded from the IEU OpenGWAS project (https://gwas.mrcieu.ac.uk/), GWAS Catalog project (https://www.ebi.ac.uk/gwas/), and ENIGMA Consortium (https://enigma.ini.usc.edu/).

## Ethics statement

This study used publicly available data from participant studies that were approved by an ethical standards committee with respect to human experimentation. No separate ethical approval was required in this study.

## Author contributions

HM and ZM proposed the idea and elaborated the research. ZM performed the main data analysis and wrote the draft of the manuscript. HM supervised the whole research and is responsible for the integrity of data analysis. All authors have given consent to the publication of this study.

## Supplementary material

Supplementary Figure 1 Scatter plots of nominal significant estimates from genetically predicted alanine transaminase on **(A)** surface area of parahippocampal gyrus; **(B)** thickness of pars opercularis; **(C)** thickness of pars orbitalis; **(D)** thickness of pericalcarine cortex. The scatter plots represented the instrument variable effects on the exposure and the outcome variables (black point), with the confidence intervals for both estimates denoted by the horizontal and vertical lines, respectively. Each colored slope was indicative of the causal effect of a unit increase in the exposure on the outcome, estimated by the method in the legend utilized to shade the trendline that was, inverse-variance weighted (light blue), weighted median (light green) and MR-Egger (dark blue).

Supplementary Figure 2 Leave-one-out plots of nominal significant estimates from genetically predicted alanine transaminase on **(A)** surface area of parahippocampal gyrus; **(B)** thickness of pars opercularis; **(C)** thickness of pars orbitalis; **(D)** thickness of pericalcarine cortex. The leave-one-out analysis was performed by recalculating the Mendelian randomization estimates using the Inverse-variance weighted estimates method, by sequentially dropping one SNP at a time to examine whether a single SNP that might have a large horizontal pleiotropic effect and influence the estimates.

Supplementary Figure 3 Funnel plots of significant and nominal significant estimates from genetically predicted alanine transaminase on **(A)** surface area of parahippocampal gyrus; **(B)** thickness of pars opercularis; **(C)** thickness of pars orbitalis; **(D)** thickness of pericalcarine cortex. Funnel plots was used to visualize overall heterogeneity of Mendelian randomization estimates for the effect of exposure on the outcomes before elimination.

Supplementary Figure 4 Scatter plots of nominal significant estimates from genetically predicted non-alcoholic fatty liver disease on **(A)** surface area of parahippocampal gyrus; **(B)** thickness of entorhinal cortex; **(C)** thickness of lateral orbitofrontal cortex; (d) thickness of pars opercularis; **(E)** thickness of pars triangularis; **(F)** thickness of pericalcarine cortex; **(G)** thickness of temporal pole. The scatter plots represented the instrument variable effects on the exposure and the outcome variables (black point), with the confidence intervals for both estimates denoted by the horizontal and vertical lines, respectively. Each colored slope was indicative of the causal effect of a unit increase in the exposure on the outcome, estimated by the method in the legend utilized to shade the trendline that was, inverse-variance weighted (light blue), weighted median (light green) and MR-Egger (dark blue).

Supplementary Figure 5 Leave-one-out plots of significant and nominal significant estimates from genetically predicted non-alcoholic fatty liver disease on **(A)** surface area of parahippocampal gyrus; **(B)** thickness of entorhinal cortex; **(C)** thickness of lateral orbitofrontal cortex; **(D)** thickness of pars opercularis; **(E)** thickness of pars triangularis; **(f)** thickness of pericalcarine cortex; **(G)** thickness of temporal pole. The leave-one-out analysis was performed by recalculating the Mendelian randomization estimates using the Inverse-variance weighted estimates method, by sequentially dropping one SNP at a time to examine whether a single SNP that might have a large horizontal pleiotropic effect and influence the estimates.

Supplementary Figure 6 Funnel plots of significant and nominal significant estimates from genetically predicted non-alcoholic fatty liver disease on **(A)** surface area of parahippocampal gyrus; **(B)** thickness of entorhinal cortex; **(C)** thickness of lateral orbitofrontal cortex; **(D)** thickness of pars opercularis; **(E)** thickness of pars triangularis; **(F)** thickness of pericalcarine cortex; **(G)** thickness of temporal pole. Funnel plots was used to visualize overall heterogeneity of Mendelian randomization estimates for the effect of exposure on the outcomes before elimination.

Supplementary Figure 7 Scatter plots of significant estimates from genetically predicted percent liver fat on **(A)** surface area of full cortex; **(B)** surface area of parahippocampal gyrus; **(C)** thickness of cuneus; **(D)** thickness of entorhinal cortex; **(E)** thickness of lingual gyrus; **(F)** thickness of pars opercularis. The scatter plots represented the instrument variable effects on the exposure and the outcome variables (black point), with the confidence intervals for both estimates denoted by the horizontal and vertical lines, respectively. Each colored slope was indicative of the causal effect of a unit increase in the exposure on the outcome, estimated by the method in the legend utilized to shade the trendline that was, inverse-variance weighted (light blue), weighted median (light green) and MR-Egger (dark blue).

Supplementary Figure 8 Leave-one-out plots of significant and nominal significant estimates from genetically predicted percent liver fat on **(A)** surface area of full cortex; **(B)** surface area of parahippocampal gyrus; **(C)** thickness of cuneus; **(D)** thickness of entorhinal cortex; **(E)** thickness of lingual gyrus; **(F)** thickness of pars opercularis. The leave-one-out analysis was performed by recalculating the Mendelian randomization estimates using the Inverse-variance weighted estimates method, by sequentially dropping one SNP at a time to examine whether a single SNP that might have a large horizontal pleiotropic effect and influence the estimates.

Supplementary Figure 9 Funnel plots of significant and nominal significant estimates from genetically predicted percent liver fat on **(A)** surface area of full cortex; **(B)** surface area of parahippocampal gyrus; **(C)** thickness of cuneus; **(D)** thickness of entorhinal cortex; **(E)** thickness of lingual gyrus; **(F)** thickness of pars opercularis. Funnel plots was used to visualize overall heterogeneity of Mendelian randomization estimates for the effect of exposure on the outcomes before elimination.

Supplementary Table 1 Case definition and exclusion criteria in the GWASs used in the present study.

Supplementary Table 2 Details about Single Nucleotide Polymorphisms used as exposures.

Supplementary Table 3 Inverse-variance weighted estimates of the effect of alanine transaminase, non-alcoholic fatty liver disease and percent liver fat on brain.

Supplementary Table 4 Inverse-variance weighted, MR-Egger and weighted median estimates of the significant and nominally significant Mendelian randomization estimates.

Supplementary Table 5 MR-PRESSO estimates of the significant and nominally significant Mendelian randomization estimates.
