# Supplementary figures and images for: Causal effects of nonalcoholic fatty liver disease on cerebral cortical structure: a Mendelian randomization analysis
Source: Front Endocrinol (Lausanne). 2023 Nov 1;14:1276576. doi: 10.3389/fendo.2023.1276576 (PMC10646496; doi:10.3389/fendo.2023.1276576)

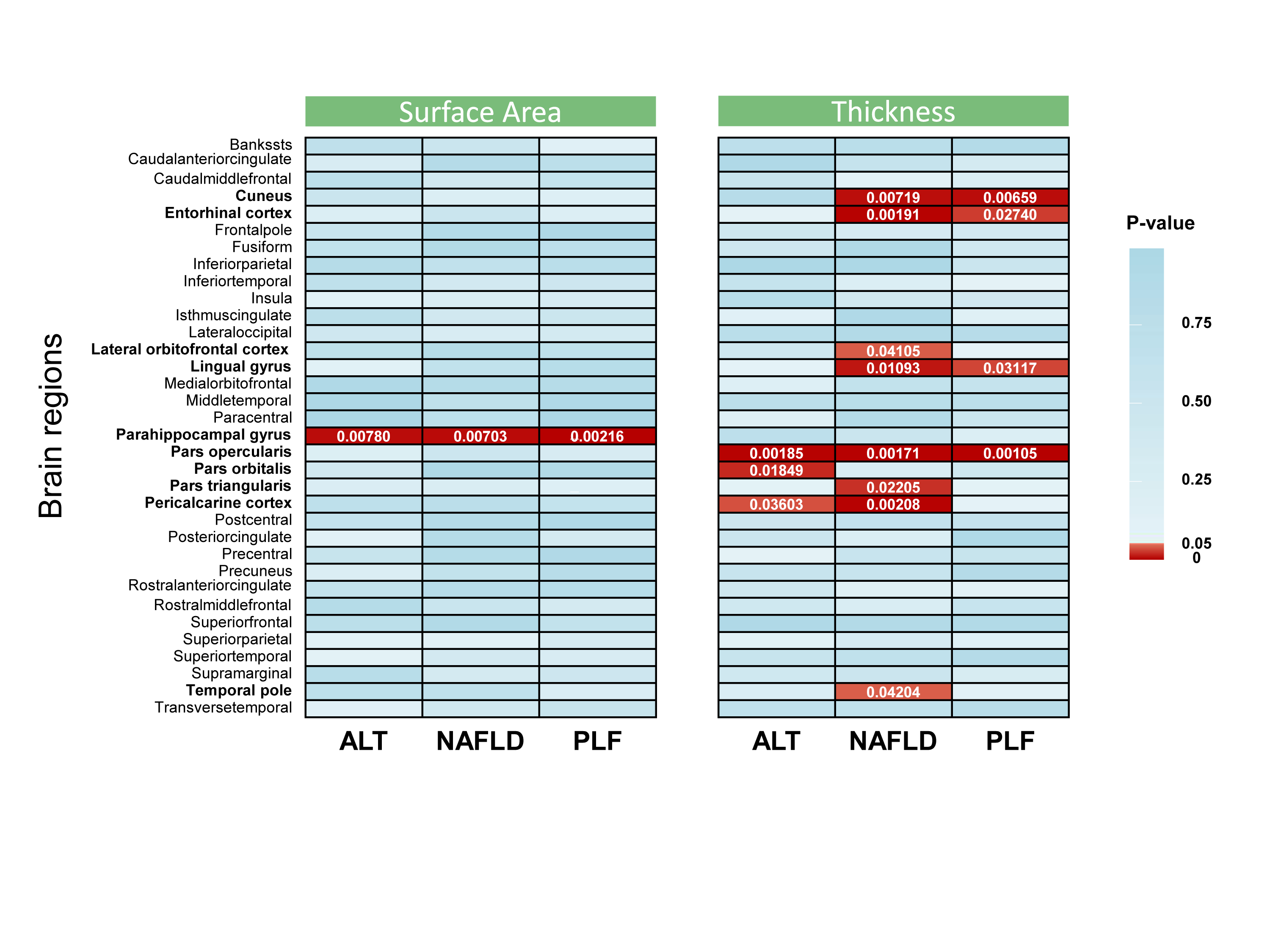

Supplement: Supplementary Figure 1 — Scatter plots of nominal significant estimates from genetically predicted alanine transaminase on (A) surface area of parahippocampal gyrus; (B) thickness of pars opercularis; (C) thickness of pars orbitalis; (D) thickness of pericalcarine cortex. The scatter plots represented the instrument variable effects on the exposure and the outcome variables (black point), with the confidence intervals for both estimates denoted by the horizontal and vertical lines, respectively. Each colored slope was indicative of the causal effect of a unit increase in the exposure on the outcome, estimated by the method in the legend utilized to shade the trendline that was, inverse-variance weighted (light blue), weighted median (light green) and MR-Egger (dark blue). [file DataSheet_1.zip › Figure_2.tif]

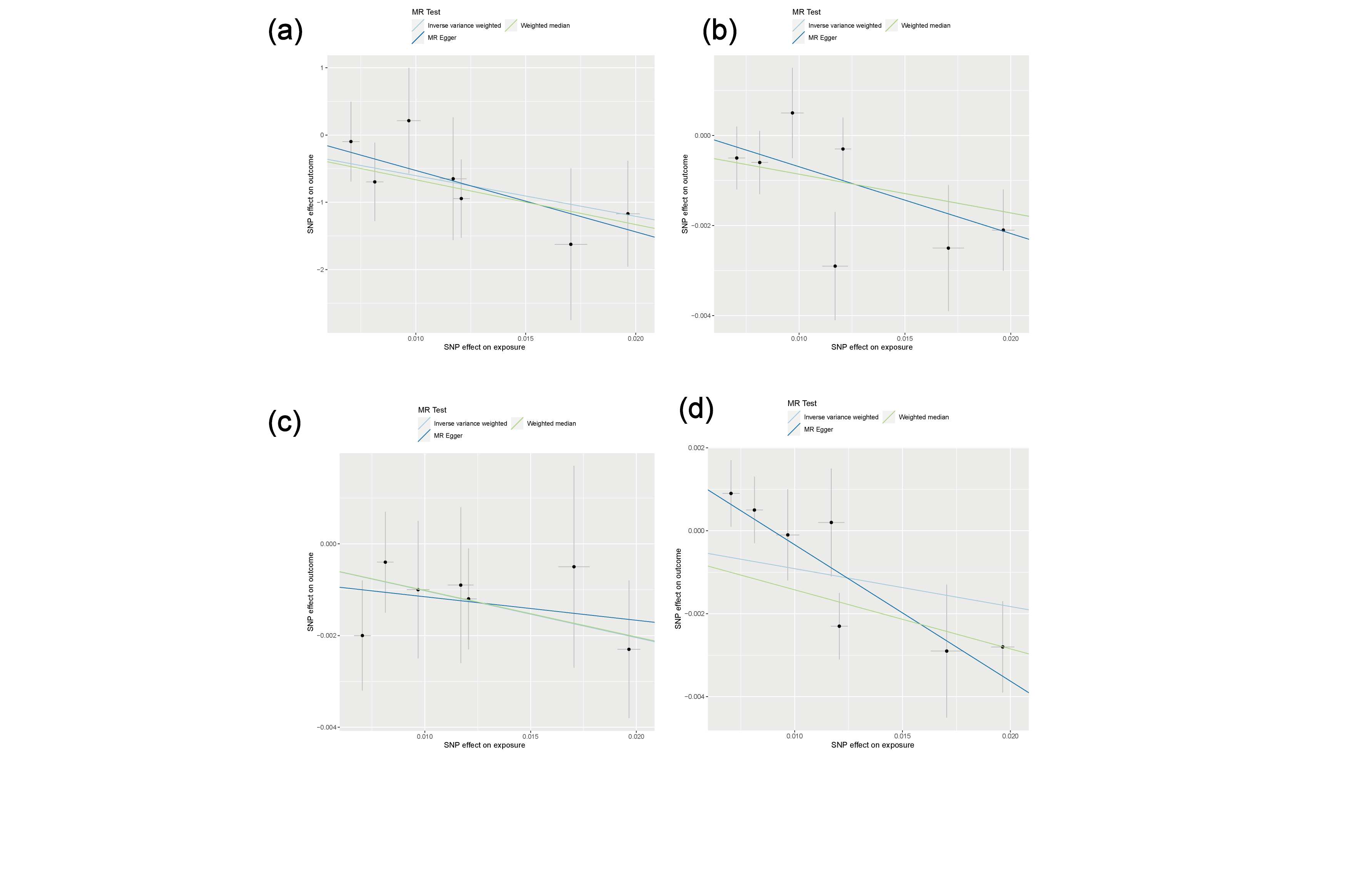

Supplement: Supplementary Figure 1 — Scatter plots of nominal significant estimates from genetically predicted alanine transaminase on (A) surface area of parahippocampal gyrus; (B) thickness of pars opercularis; (C) thickness of pars orbitalis; (D) thickness of pericalcarine cortex. The scatter plots represented the instrument variable effects on the exposure and the outcome variables (black point), with the confidence intervals for both estimates denoted by the horizontal and vertical lines, respectively. Each colored slope was indicative of the causal effect of a unit increase in the exposure on the outcome, estimated by the method in the legend utilized to shade the trendline that was, inverse-variance weighted (light blue), weighted median (light green) and MR-Egger (dark blue). [file DataSheet_1.zip › Figure_S1.tif]

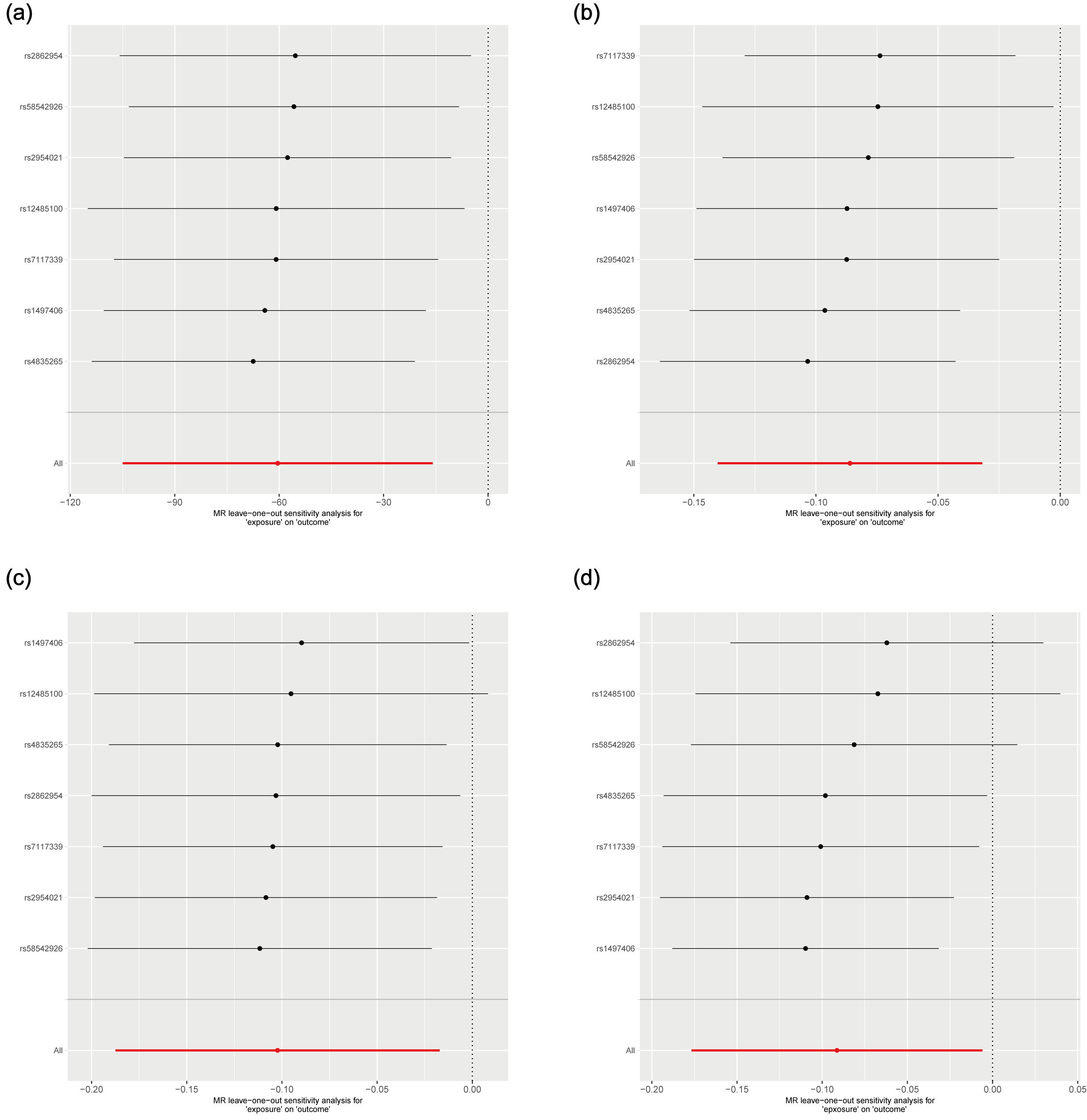

Supplement: Supplementary Figure 1 — Scatter plots of nominal significant estimates from genetically predicted alanine transaminase on (A) surface area of parahippocampal gyrus; (B) thickness of pars opercularis; (C) thickness of pars orbitalis; (D) thickness of pericalcarine cortex. The scatter plots represented the instrument variable effects on the exposure and the outcome variables (black point), with the confidence intervals for both estimates denoted by the horizontal and vertical lines, respectively. Each colored slope was indicative of the causal effect of a unit increase in the exposure on the outcome, estimated by the method in the legend utilized to shade the trendline that was, inverse-variance weighted (light blue), weighted median (light green) and MR-Egger (dark blue). [file DataSheet_1.zip › Figure_S2.tif]

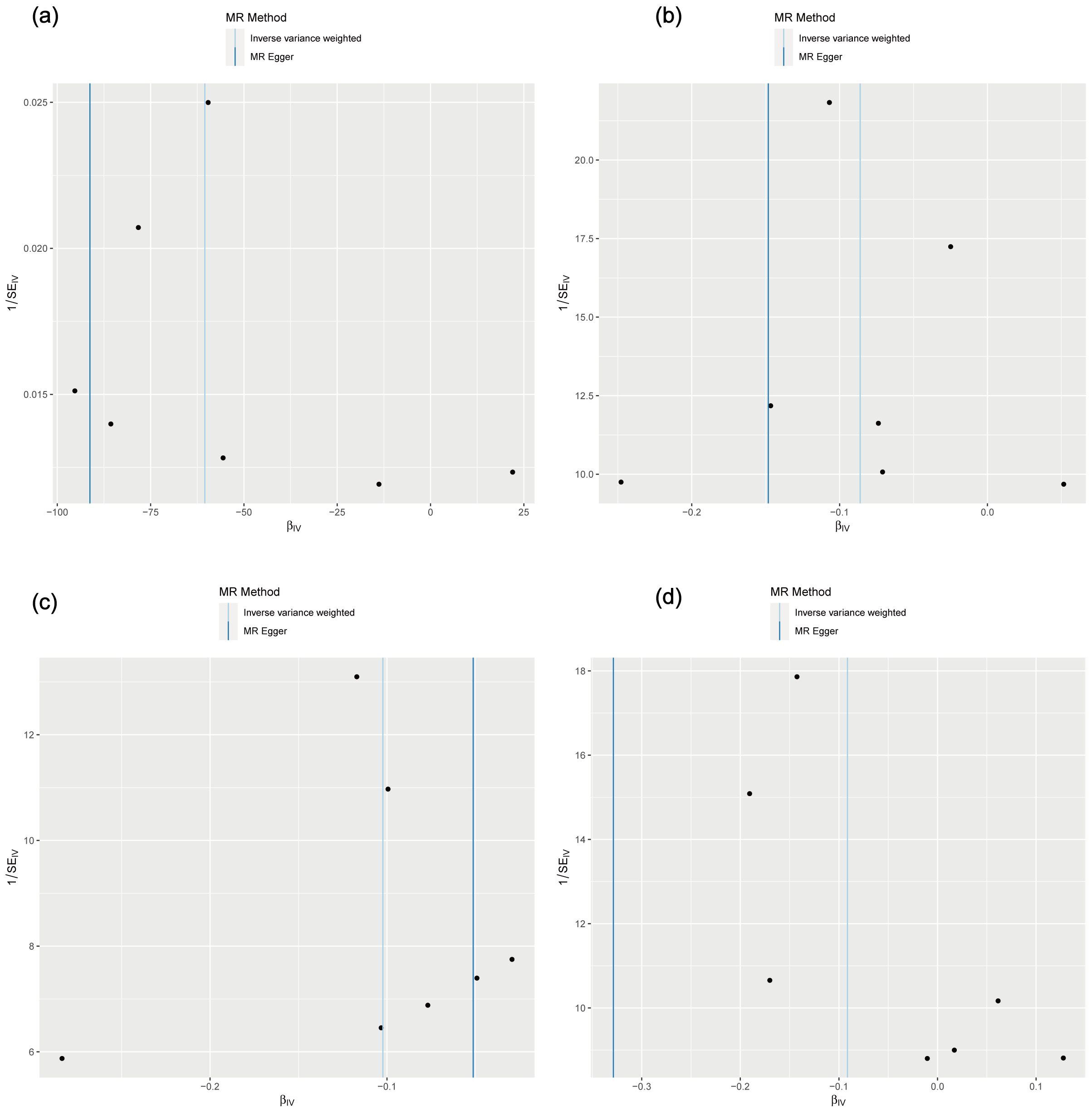

Supplement: Supplementary Figure 1 — Scatter plots of nominal significant estimates from genetically predicted alanine transaminase on (A) surface area of parahippocampal gyrus; (B) thickness of pars opercularis; (C) thickness of pars orbitalis; (D) thickness of pericalcarine cortex. The scatter plots represented the instrument variable effects on the exposure and the outcome variables (black point), with the confidence intervals for both estimates denoted by the horizontal and vertical lines, respectively. Each colored slope was indicative of the causal effect of a unit increase in the exposure on the outcome, estimated by the method in the legend utilized to shade the trendline that was, inverse-variance weighted (light blue), weighted median (light green) and MR-Egger (dark blue). [file DataSheet_1.zip › Figure_S3.tif]

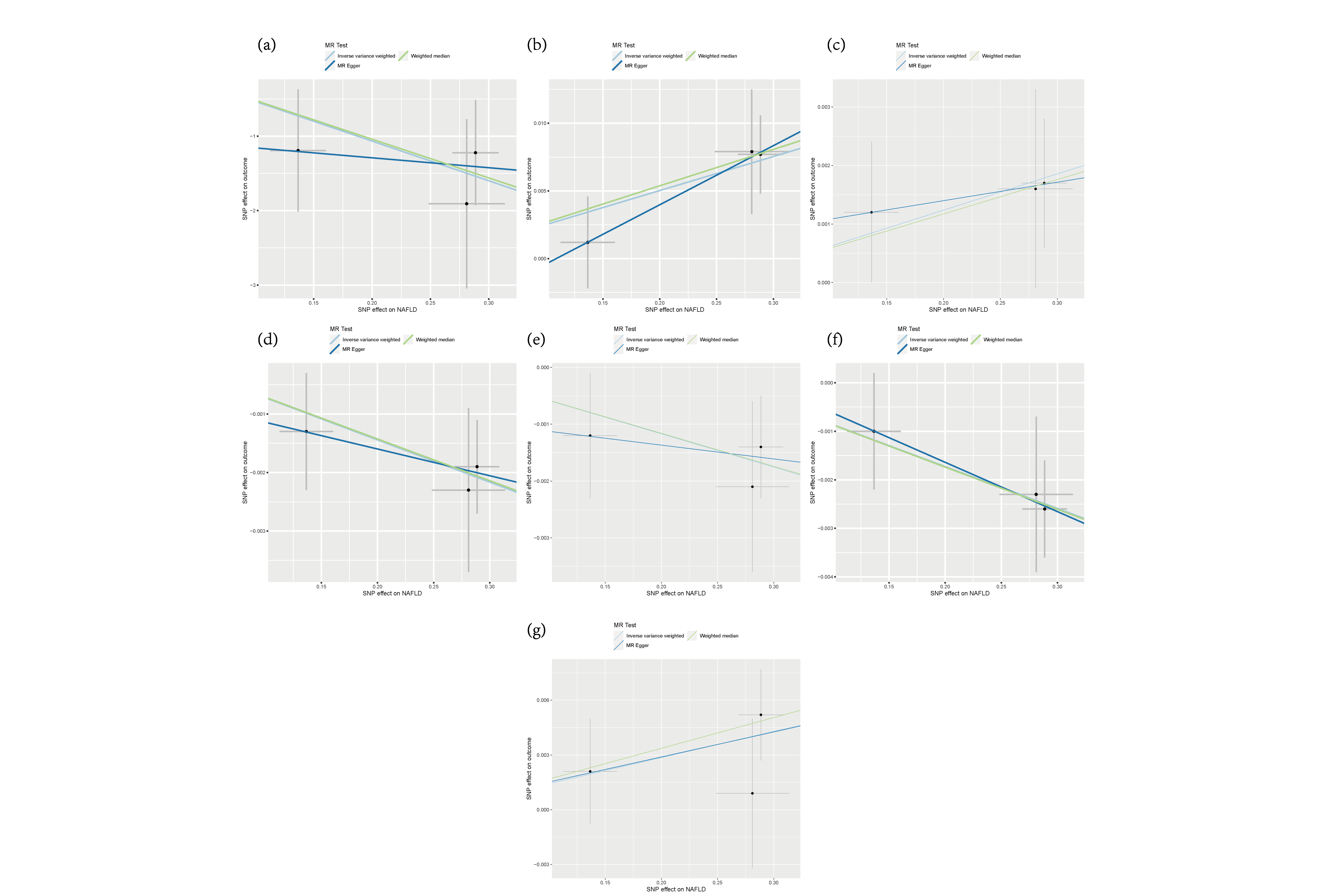

Supplement: Supplementary Figure 1 — Scatter plots of nominal significant estimates from genetically predicted alanine transaminase on (A) surface area of parahippocampal gyrus; (B) thickness of pars opercularis; (C) thickness of pars orbitalis; (D) thickness of pericalcarine cortex. The scatter plots represented the instrument variable effects on the exposure and the outcome variables (black point), with the confidence intervals for both estimates denoted by the horizontal and vertical lines, respectively. Each colored slope was indicative of the causal effect of a unit increase in the exposure on the outcome, estimated by the method in the legend utilized to shade the trendline that was, inverse-variance weighted (light blue), weighted median (light green) and MR-Egger (dark blue). [file DataSheet_1.zip › Figure_S4.tif]

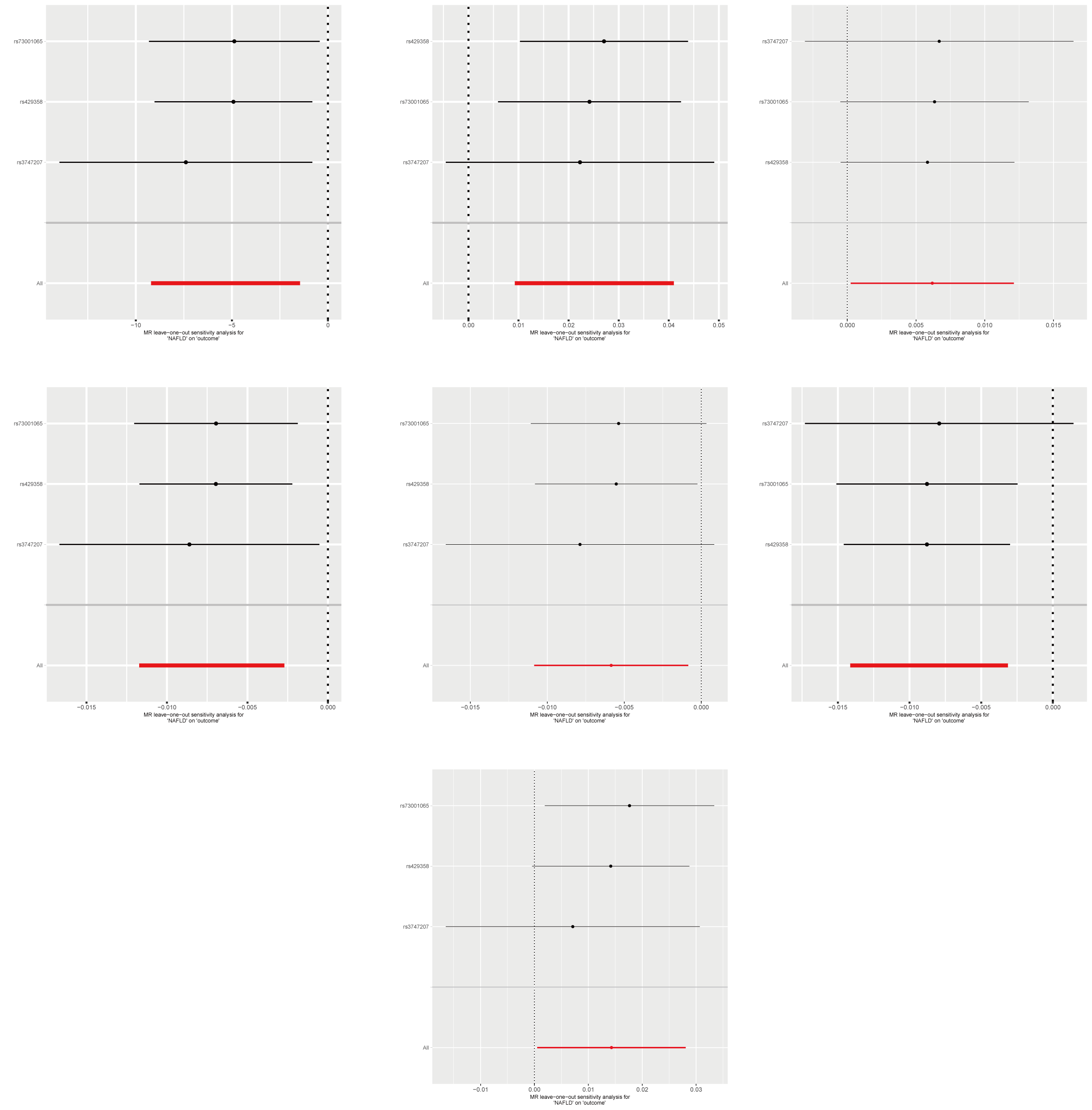

Supplement: Supplementary Figure 1 — Scatter plots of nominal significant estimates from genetically predicted alanine transaminase on (A) surface area of parahippocampal gyrus; (B) thickness of pars opercularis; (C) thickness of pars orbitalis; (D) thickness of pericalcarine cortex. The scatter plots represented the instrument variable effects on the exposure and the outcome variables (black point), with the confidence intervals for both estimates denoted by the horizontal and vertical lines, respectively. Each colored slope was indicative of the causal effect of a unit increase in the exposure on the outcome, estimated by the method in the legend utilized to shade the trendline that was, inverse-variance weighted (light blue), weighted median (light green) and MR-Egger (dark blue). [file DataSheet_1.zip › Figure_S5.tif]

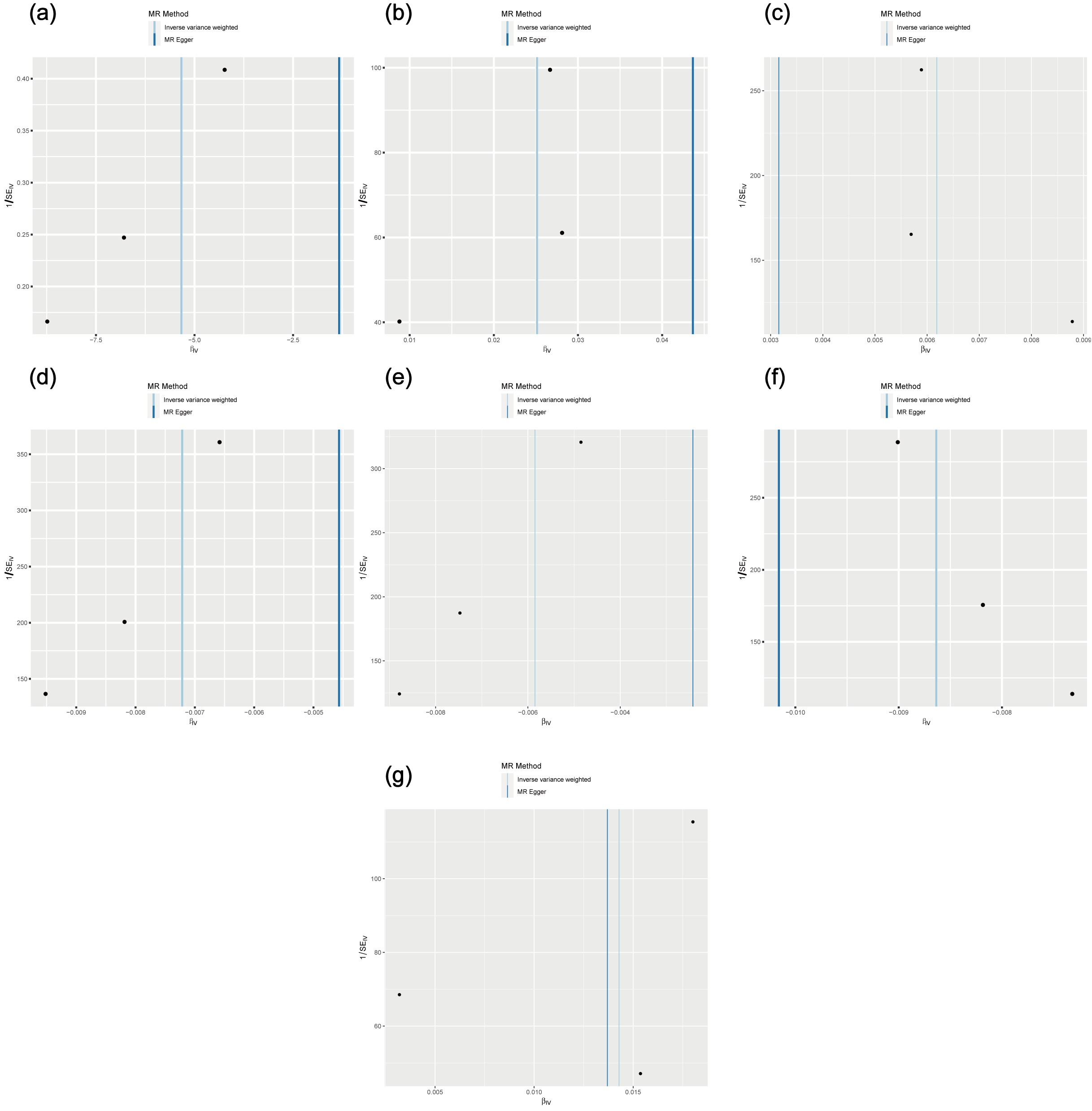

Supplement: Supplementary Figure 1 — Scatter plots of nominal significant estimates from genetically predicted alanine transaminase on (A) surface area of parahippocampal gyrus; (B) thickness of pars opercularis; (C) thickness of pars orbitalis; (D) thickness of pericalcarine cortex. The scatter plots represented the instrument variable effects on the exposure and the outcome variables (black point), with the confidence intervals for both estimates denoted by the horizontal and vertical lines, respectively. Each colored slope was indicative of the causal effect of a unit increase in the exposure on the outcome, estimated by the method in the legend utilized to shade the trendline that was, inverse-variance weighted (light blue), weighted median (light green) and MR-Egger (dark blue). [file DataSheet_1.zip › Figure_S6.tif]

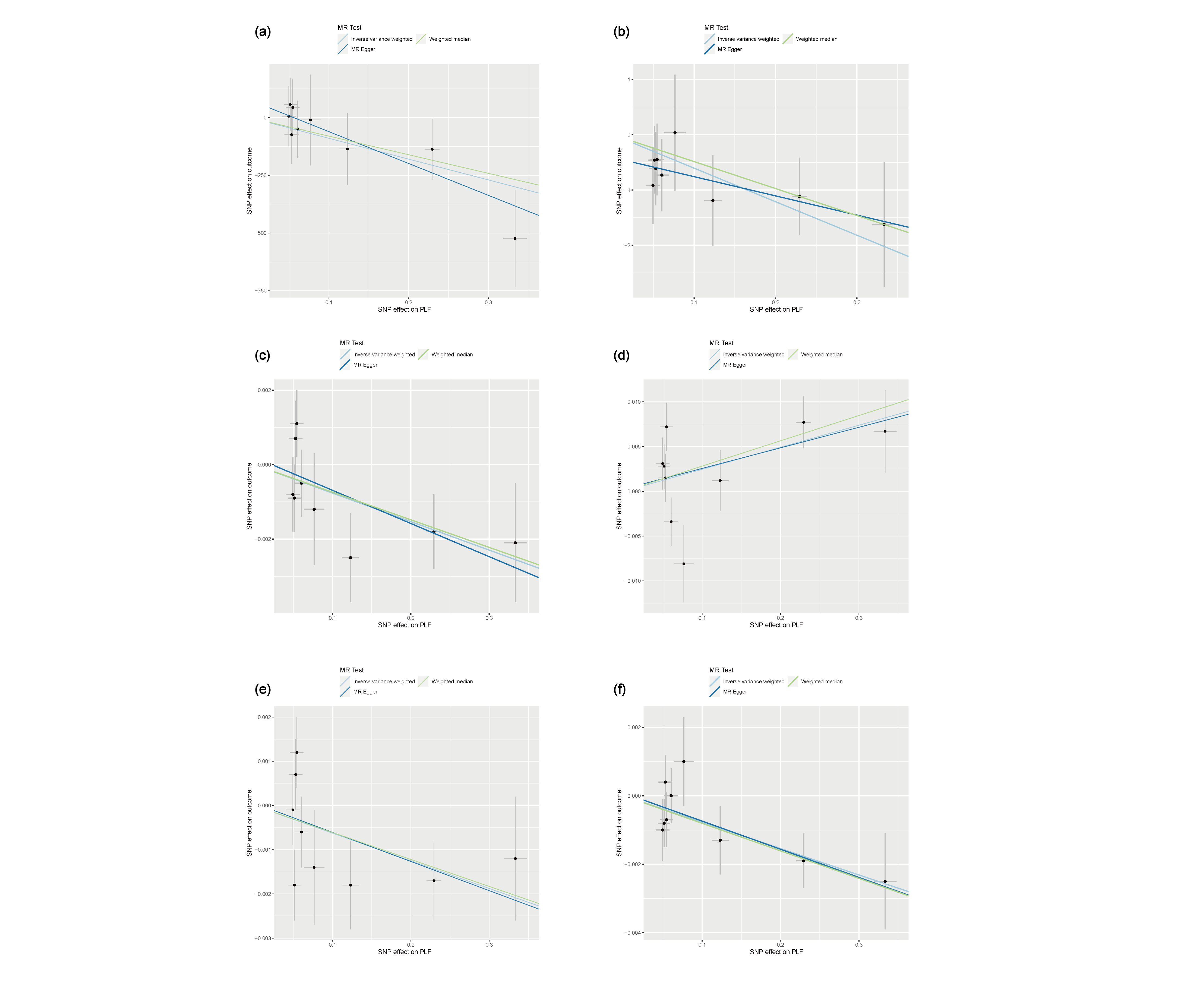

Supplement: Supplementary Figure 1 — Scatter plots of nominal significant estimates from genetically predicted alanine transaminase on (A) surface area of parahippocampal gyrus; (B) thickness of pars opercularis; (C) thickness of pars orbitalis; (D) thickness of pericalcarine cortex. The scatter plots represented the instrument variable effects on the exposure and the outcome variables (black point), with the confidence intervals for both estimates denoted by the horizontal and vertical lines, respectively. Each colored slope was indicative of the causal effect of a unit increase in the exposure on the outcome, estimated by the method in the legend utilized to shade the trendline that was, inverse-variance weighted (light blue), weighted median (light green) and MR-Egger (dark blue). [file DataSheet_1.zip › Figure_S7.tif]

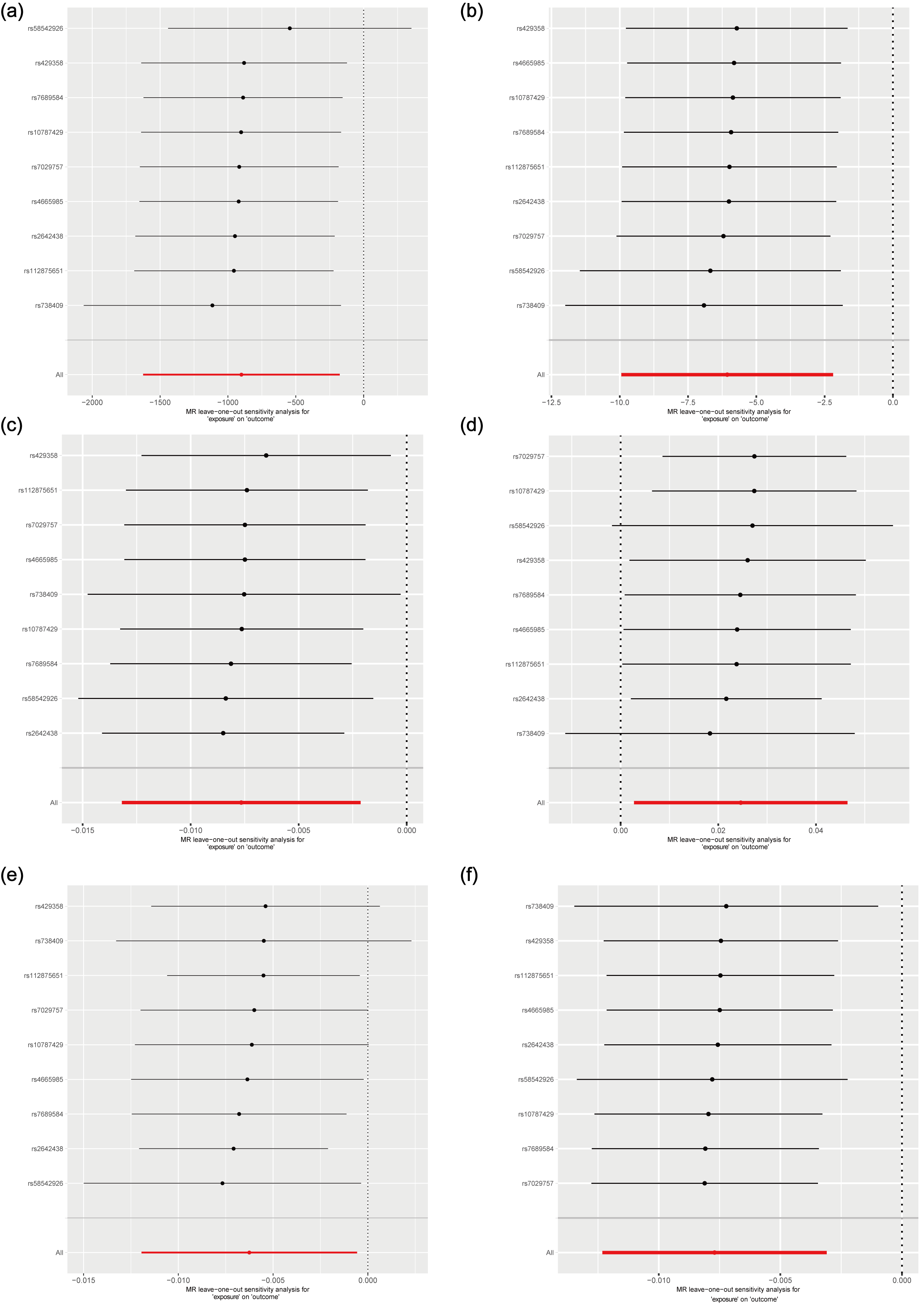

Supplement: Supplementary Figure 1 — Scatter plots of nominal significant estimates from genetically predicted alanine transaminase on (A) surface area of parahippocampal gyrus; (B) thickness of pars opercularis; (C) thickness of pars orbitalis; (D) thickness of pericalcarine cortex. The scatter plots represented the instrument variable effects on the exposure and the outcome variables (black point), with the confidence intervals for both estimates denoted by the horizontal and vertical lines, respectively. Each colored slope was indicative of the causal effect of a unit increase in the exposure on the outcome, estimated by the method in the legend utilized to shade the trendline that was, inverse-variance weighted (light blue), weighted median (light green) and MR-Egger (dark blue). [file DataSheet_1.zip › Figure_S8.tif]

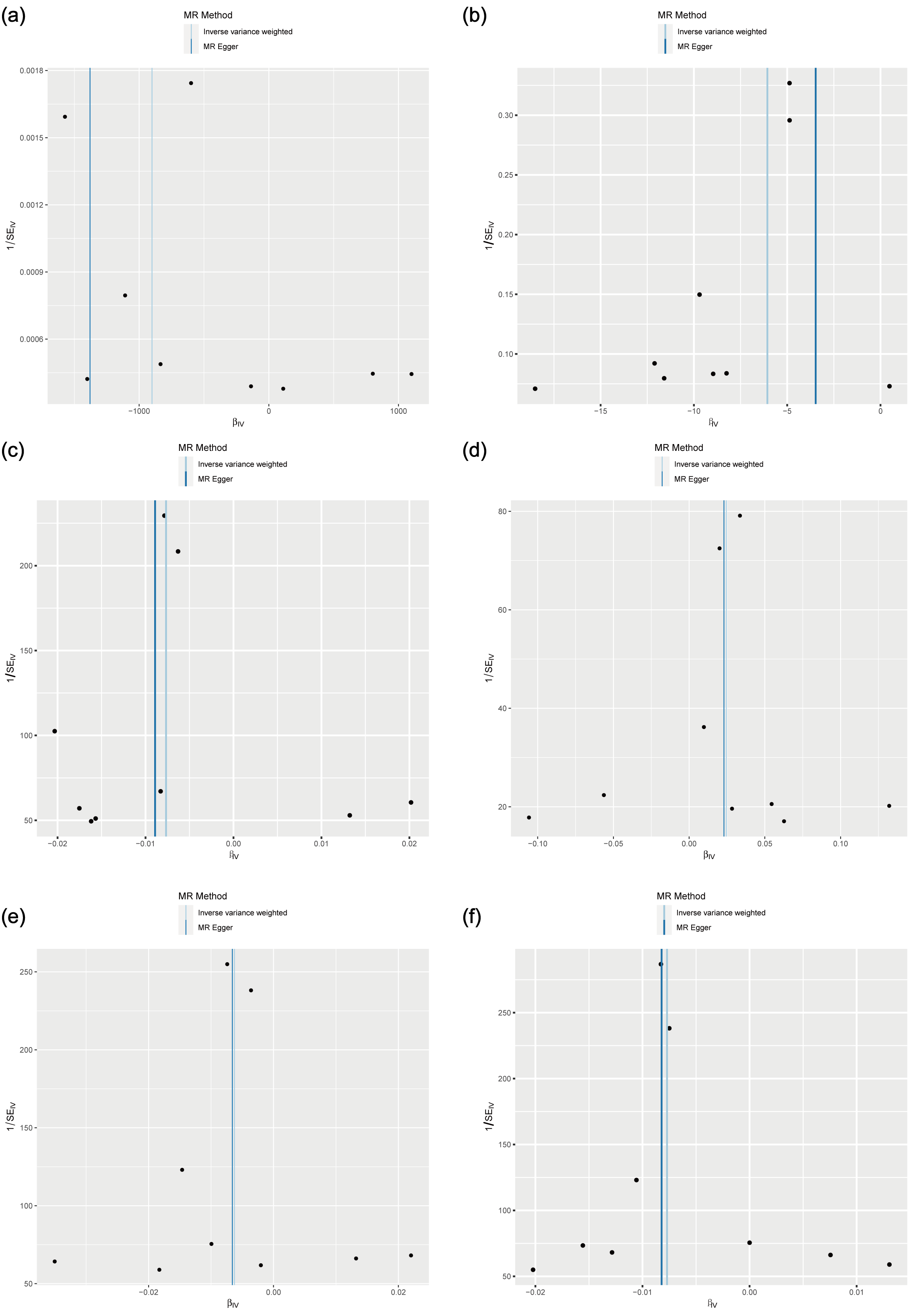

Supplement: Supplementary Figure 1 — Scatter plots of nominal significant estimates from genetically predicted alanine transaminase on (A) surface area of parahippocampal gyrus; (B) thickness of pars opercularis; (C) thickness of pars orbitalis; (D) thickness of pericalcarine cortex. The scatter plots represented the instrument variable effects on the exposure and the outcome variables (black point), with the confidence intervals for both estimates denoted by the horizontal and vertical lines, respectively. Each colored slope was indicative of the causal effect of a unit increase in the exposure on the outcome, estimated by the method in the legend utilized to shade the trendline that was, inverse-variance weighted (light blue), weighted median (light green) and MR-Egger (dark blue). [file DataSheet_1.zip › Figure_S9.tif]
