# Supplementary material for: Causal effects of nonalcoholic fatty liver disease on cerebral cortical structure: a Mendelian randomization analysis
Source: Front Endocrinol (Lausanne). 2023 Nov 1;14:1276576. doi: 10.3389/fendo.2023.1276576 (PMC10646496; doi:10.3389/fendo.2023.1276576)
Supplement: Supplementary Table 1 — Case definition and exclusion criteria in the GWASs used in the present study. [file Table_1.docx]

**Table S1.** Case definition and exclusion criteria in the GWASs used in the present study

| **GWAS data source** | **Study** | **Sample size or case/control** | **Cases definition** | **Exclusion criteria** |
| --- | --- | --- | --- | --- |
| ALT | PMID: 33972514 | 437,267 | None | Withdrawn consent participants, Pregnant or uncertain participants, Non-European participants |
| NAFLD | eMERGE | 1,106/8,571 | ICD-9: 571.5, ICD-9: 571.8, ICD-9: 571.9, ICD-10: K75.81, ICD-10: K76.0 and ICD-10: K76.9 | Alcohol dependence, alcoholic liver disease, alpha-1 antitrypsin deficiency, Alagille syndrome, liver transplant, cystic fibrosis, hepatitis, abetalipoproteinemia, rare autosomal recessive disease, lipodystrophy, disorders of copper metabolism Reye’s syndrome, inborn errors of metabolism, HELLP syndrome, starvation and acute fatty liver. |
|  | UK Biobank | 2,558/395,241 | ICD-10: K74.0 and K74.2 (hepatic fibrosis), K75.8 (NASH), K76.0 (NAFLD) and ICD-10: K76.9 (other specified diseases of the liver) | Same as eMERGE |
|  | Estonian Biobank | 4,119/190,120 | ICD-10: K74.0 and K74.2 (hepatic fibrosis), K75.8 (NASH), K76.0 (NAFLD) and ICD-10: K76.9 (other specified diseases of the liver) | Same as eMERGE |
|  | FinnGen | 651/176,248 | ICD-10: K76.0 (NAFLD) | None |
| PLF | PMID: 34128465 | 32,858 | None | None |
| SA and TH of Brain region | ENIGMA Consortium | 51,665 | None | None |

**ALT**, alanine transaminase; **HER**; electronic health record; **eMERGE**; Electronic Medical Records and Genomics; **GWAS**; genome-wide association study; **ICD**, International Classification of Diseases; **NAFLD**, non-alcoholic fatty liver disease; **PLF**, percent liver fat; **SA**, surface area; **TH**, thickness.
