# Supplementary material for: Causal effects of nonalcoholic fatty liver disease on cerebral cortical structure: a Mendelian randomization analysis
Source: Front Endocrinol (Lausanne). 2023 Nov 1;14:1276576. doi: 10.3389/fendo.2023.1276576 (PMC10646496; doi:10.3389/fendo.2023.1276576)
Supplement: Supplementary Table 2 — Details about Single Nucleotide Polymorphisms used as exposures. [file Table_2.docx]

**Table S2.** Details about Single Nucleotide Polymorphisms used as exposures

| **SNP** | **Beta** | **SE** | **P value** | **Effect allele** | **Other allele** | **EAF** | **Phenotype** | **F statistic** |
| --- | --- | --- | --- | --- | --- | --- | --- | --- |
| rs1497406 | 0.00705 | 0.000392 | 4.70E-72 | G | A | 0.579183 | ALT | 10.6 |
| rs71633359 | -0.0089 | 0.000427 | 9.80E-97 | C | T | 0.318358 | ALT | 15 |
| rs4835265 | 0.009682 | 0.000532 | 7.70E-76 | A | C | 0.158115 | ALT | 10.9 |
| rs2954021 | -0.00813 | 0.000389 | 4.50E-98 | G | A | 0.504969 | ALT | 14.5 |
| rs112574791 | -0.04081 | 0.001727 | 3.60E-124 | A | G | 0.01281 | ALT | 18.4 |
| rs7041363 | -0.00957 | 0.000391 | 1.40E-136 | G | C | 0.488074 | ALT | 20 |
| rs2862954 | -0.01207 | 0.000389 | 1.00E-200 | C | T | 0.49717 | ALT | 31.8 |
| rs7117339 | -0.0117 | 0.000606 | 1.80E-83 | T | C | 0.116603 | ALT | 12.3 |
| rs58542926 | 0.01705 | 0.000739 | 1.80E-119 | T | C | 0.074762 | ALT | 17.6 |
| rs12485100 | 0.019647 | 0.000534 | 1.00E-200 | T | G | 0.157076 | ALT | 44.7 |
| rs2642438 | 0.054495 | 0.008419 | 9.60E-11 | G | A | 0.7043 | PLF | 40.7 |
| rs4665985 | 0.049457 | 0.008739 | 1.50E-08 | C | A | 0.270167 | PLF | 31.7 |
| rs7689584 | -0.05294 | 0.008816 | 1.90E-09 | G | T | 0.253933 | PLF | 34.9 |
| rs112875651 | -0.05138 | 0.007931 | 9.30E-11 | A | G | 0.394576 | PLF | 41.5 |
| rs7029757 | -0.07659 | 0.013083 | 4.80E-09 | A | G | 0.096424 | PLF | 33.6 |
| rs10787429 | -0.06039 | 0.008644 | 2.80E-12 | C | T | 0.727416 | PLF | 47.6 |
| rs188247550 | 0.336267 | 0.033357 | 6.70E-24 | T | C | 0.01458 | PLF | 107.1 |
| rs58542926 | 0.333425 | 0.014543 | 2.50E-116 | T | C | 0.075455 | PLF | 517.7 |
| rs429358 | -0.12303 | 0.010679 | 1.00E-30 | C | T | 0.152548 | PLF | 129.1 |
| rs738409 | 0.229457 | 0.00933 | 1.50E-133 | G | C | 0.213803 | PLF | 592 |
| rs28601761 | 0.110033 | 0.016443 | 2.20E-11 | C | G | 0.592445 | NAFLD | 4579.1 |
| rs73001065 | 0.281009 | 0.032641 | 7.36E-18 | C | G | 0.063618 | NAFLD | 7394.9 |
| rs429358 | 0.136615 | 0.023929 | 1.14E-08 | T | C | 0.84493 | NAFLD | 3826.7 |
| rs3747207 | 0.288601 | 0.019822 | 5.07E-48 | A | G | 0.222664 | NAFLD | 23115.9 |

**ALT**, alanine transaminase; **EAF**, effect allele frequency; **NAFLD**, non-alcoholic fatty liver disease; **PLF**, percent liver fat; **SE**, Standard error; **SNP**, Single Nucleotide Polymorphisms.
