# Supplementary material for: Causal effects of nonalcoholic fatty liver disease on cerebral cortical structure: a Mendelian randomization analysis
Source: Front Endocrinol (Lausanne). 2023 Nov 1;14:1276576. doi: 10.3389/fendo.2023.1276576 (PMC10646496; doi:10.3389/fendo.2023.1276576)
Supplement: Supplementary Table 3 — Inverse-variance weighted estimates of the effect of alanine transaminase, non-alcoholic fatty liver disease and percent liver fat on brain. [file Table_3.docx]

**Table S3.** Inverse-variance weighted estimates of the effect of alanine transaminase, non-alcoholic fatty liver disease and percent liver fat on brain

| **Exposures**  **Outcomes** | **Method** | **β (95%CI)** | **SE** | **P value** |
| --- | --- | --- | --- | --- |
| **ALT** |  |  |  |  |
| Surface area of bankssts | IVW | -14.235(-85.2122, 56.7422) | 36.21286 | 0.69425 |
| Surface area of caudalanteriorcingulate | IVW | 31.9656(-25.6837, 89.6149) | 29.41292 | 0.27713 |
| Surface area of caudalmiddlefrontal | IVW | 28.4997(-123.6381, 180.6375) | 77.62133 | 0.7135 |
| Surface area of cuneus | IVW | 29.5372(-58.8387, 117.9131) | 45.08975 | 0.51242 |
| Surface area of entorhinal | IVW | -28.199(-73.7878, 17.3898) | 23.25961 | 0.22538 |
| Surface area of frontalpole | IVW | -8.0682(-33.3627, 17.2264) | 12.90538 | 0.53185 |
| Surface area of fusiform | IVW | 34.4281(-110.2554, 179.1117) | 73.81815 | 0.64094 |
| Surface area of inferiorparietal | IVW | -27.1296(-274.2601, 220.0009) | 126.08699 | 0.82964 |
| Surface area of inferiortemporal | IVW | 54.7868(-219.4949, 329.0685) | 139.93966 | 0.69543 |
| Surface area of insula | IVW | 78.8832(-19.6945, 177.4609) | 50.29473 | 0.11678 |
| Surface area of isthmuscingulate | IVW | 16.3132(-77.2333, 109.8597) | 47.7278 | 0.7325 |
| Surface area of lateraloccipital | IVW | 108.2262(-160.7988, 377.2511) | 137.25761 | 0.43041 |
| Surface area of lateralorbitofrontal | IVW | 21.1106(-85.2334, 127.4546) | 54.25713 | 0.69721 |
| Surface area of lingual | IVW | -188.0849(-423.5774, 47.4076) | 120.14925 | 0.11748 |
| Surface area of medialorbitofrontal | IVW | 3.0982(-75.8185, 82.015) | 40.26366 | 0.93866 |
| Surface area of middletemporal | IVW | -2.2125(-143.8251, 139.4002) | 72.25136 | 0.97557 |
| Surface area of paracentral | IVW | -0.179(-135.8132, 135.4551) | 69.20107 | 0.99794 |
| Surface area of parahippocampal | IVW | -60.4594(-104.9948, -15.9239) | 22.72216 | 0.0078 |
| Surface area of parsopercularis | IVW | -66.9854(-168.7999, 34.829) | 51.94615 | 0.19722 |
| Surface area of parsorbitalis | IVW | -15.2686(-50.9892, 20.4521) | 18.22484 | 0.40215 |
| Surface area of parstriangularis | IVW | 56.8701(-37.5957, 151.336) | 48.19688 | 0.23802 |
| Surface area of pericalcarine | IVW | -27.2759(-172.0303, 117.4786) | 73.85432 | 0.71189 |
| Surface area of postcentral | IVW | 43.7047(-120.6208, 208.0302) | 83.83954 | 0.60216 |
| Surface area of posteriorcingulate | IVW | 55.2868(-14.0759, 124.6495) | 35.38911 | 0.11823 |
| Surface area of precentral | IVW | 57.7658(-135.5346, 251.0663) | 98.62267 | 0.55806 |
| Surface area of precuneus | IVW | -105.897(-271.0539, 59.2598) | 84.2637 | 0.20885 |
| Surface area of rostralanteriorcingulate | IVW | 14.2928(-41.6445, 70.2302) | 28.53947 | 0.61651 |
| Surface area of rostralmiddlefrontal | IVW | -29.9612(-331.7561, 271.8337) | 153.977 | 0.84572 |
| Surface area of superiorfrontal | IVW | 38.2228(-194.1659, 270.6116) | 118.56568 | 0.74717 |
| Surface area of superiorparietal | IVW | -185.3877(-421.2867, 50.5113) | 120.35663 | 0.12348 |
| Surface area of superiortemporal | IVW | 119.0068(-17.9322, 255.9459) | 69.86685 | 0.0885 |
| Surface area of supramarginal | IVW | -25.3738(-227.1846, 176.4371) | 102.96474 | 0.80535 |
| Surface area of temporalpole | IVW | 6.37(-25.8314, 38.5714) | 16.42929 | 0.69822 |
| Surface area of transversetemporal | IVW | 25.8016(-8.6201, 60.2233) | 17.56209 | 0.14179 |
| Thickness of bankssts | IVW | 0.0199(-0.0901, 0.1299) | 0.05613 | 0.72289 |
| Thickness of caudalanteriorcingulate | IVW | 0.0061(-0.1442, 0.1563) | 0.07665 | 0.93706 |
| Thickness of caudalmiddlefrontal | IVW | -0.0166(-0.072, 0.0388) | 0.02827 | 0.55771 |
| Thickness of cuneus | IVW | -0.0115(-0.1073, 0.0843) | 0.04888 | 0.81449 |
| Thickness of entorhinal | IVW | 0.191(-0.0192, 0.4012) | 0.10723 | 0.07493 |
| Thickness of frontalpole | IVW | -0.0505(-0.1793, 0.0783) | 0.06573 | 0.44225 |
| Thickness of fusiform | IVW | 0.0243(-0.0368, 0.0854) | 0.03117 | 0.43501 |
| Thickness of inferiorparietal | IVW | 0.0005(-0.0962, 0.0973) | 0.04936 | 0.99182 |
| Thickness of inferiortemporal | IVW | -0.0195(-0.0884, 0.0495) | 0.03518 | 0.58014 |
| Thickness of insula | IVW | 0.0096(-0.0612, 0.0804) | 0.03612 | 0.79072 |
| Thickness of isthmuscingulate | IVW | -0.1066(-0.2476, 0.0344) | 0.07193 | 0.13831 |
| Thickness of lateraloccipital | IVW | 0.008(-0.0442, 0.0601) | 0.02662 | 0.76451 |
| Thickness of lateralorbitofrontal | IVW | -0.0273(-0.0975, 0.0428) | 0.0358 | 0.44513 |
| Thickness of lingual | IVW | -0.052(-0.1074, 0.0034) | 0.02827 | 0.06602 |
| Thickness of medialorbitofrontal | IVW | -0.0531(-0.1311, 0.0248) | 0.03976 | 0.1814 |
| Thickness of middletemporal | IVW | -0.0106(-0.0731, 0.0519) | 0.03189 | 0.73876 |
| Thickness of paracentral | IVW | 0.0559(-0.025, 0.1368) | 0.04125 | 0.17536 |
| Thickness of parahippocampal | IVW | 0.0462(-0.2045, 0.297) | 0.12792 | 0.71769 |
| Thickness of parsopercularis | IVW | -0.0861(-0.1402, -0.0319) | 0.02764 | 0.00185 |
| Thickness of parsorbitalis | IVW | -0.1023(-0.1874, -0.0172) | 0.04343 | 0.01849 |
| Thickness of parstriangularis | IVW | -0.0505(-0.1093, 0.0082) | 0.02998 | 0.09184 |
| Thickness of pericalcarine | IVW | -0.0913(-0.1767, -0.006) | 0.04356 | 0.03603 |
| Thickness of postcentral | IVW | 0.0193(-0.0324, 0.0711) | 0.0264 | 0.46417 |
| Thickness of posteriorcingulate | IVW | 0.0239(-0.0443, 0.0921) | 0.03478 | 0.49222 |
| Thickness of precentral | IVW | 0.0527(-0.0052, 0.1107) | 0.02958 | 0.07464 |
| Thickness of precuneus | IVW | 0.014(-0.032, 0.06) | 0.02347 | 0.54982 |
| Thickness of rostralanteriorcingulate | IVW | 0.0415(-0.0638, 0.1469) | 0.05376 | 0.43962 |
| Thickness of rostralmiddlefrontal | IVW | -0.02(-0.0687, 0.0287) | 0.02484 | 0.42013 |
| Thickness of superiorfrontal | IVW | 0.002(-0.0508, 0.0548) | 0.02693 | 0.94014 |
| Thickness of superiorparietal | IVW | -0.0365(-0.082, 0.009) | 0.02323 | 0.11609 |
| Thickness of superiortemporal | IVW | -0.0207(-0.0897, 0.0484) | 0.03525 | 0.55788 |
| Thickness of supramarginal | IVW | 0.0208(-0.0299, 0.0716) | 0.0259 | 0.42147 |
| Thickness of temporalpole | IVW | 0.1215(-0.0801, 0.323) | 0.10284 | 0.23754 |
| Thickness of transversetemporal | IVW | -0.0282(-0.1544, 0.098) | 0.06441 | 0.66145 |
| **NAFLD** |  |  |  |  |
| Surface area of bankssts | IVW | -2.094(-8.3656, 4.1775) | 3.19977 | 0.51283 |
| Surface area of caudalanteriorcingulate | IVW | 0.8216(-7.8497, 9.4929) | 4.42413 | 0.85268 |
| Surface area of caudalmiddlefrontal | IVW | 5.6666(-7.577, 18.9102) | 6.75693 | 0.40167 |
| Surface area of cuneus | IVW | 5.7785(-3.2339, 14.791) | 4.59819 | 0.20886 |
| Surface area of entorhinal | IVW | -2.096(-8.2296, 4.0376) | 3.12939 | 0.503 |
| Surface area of frontalpole | IVW | -0.1236(-1.622, 1.3748) | 0.76448 | 0.87155 |
| Surface area of fusiform | IVW | 0.9461(-11.6524, 13.5445) | 6.42778 | 0.88298 |
| Surface area of inferiorparietal | IVW | 4.8069(-16.708, 26.3218) | 10.977 | 0.66145 |
| Surface area of inferiortemporal | IVW | 12.5801(-15.5179, 40.678) | 14.33569 | 0.3802 |
| Surface area of insula | IVW | 6.2481(-4.2764, 16.7726) | 5.36966 | 0.24459 |
| Surface area of isthmuscingulate | IVW | -2.4865(-8.2564, 3.2833) | 2.94379 | 0.39829 |
| Surface area of lateraloccipital | IVW | 15.3225(-11.9312, 42.5762) | 13.90494 | 0.27048 |
| Surface area of lateralorbitofrontal | IVW | -0.9643(-10.7018, 8.7733) | 4.96814 | 0.8461 |
| Surface area of lingual | IVW | -5.184(-27.5711, 17.2032) | 11.42201 | 0.64993 |
| Surface area of medialorbitofrontal | IVW | -0.6667(-7.5337, 6.2003) | 3.50357 | 0.84909 |
| Surface area of middletemporal | IVW | 2.6744(-10.0797, 15.4285) | 6.5072 | 0.68108 |
| Surface area of paracentral | IVW | 2.2395(-9.8146, 14.2936) | 6.15005 | 0.71575 |
| Surface area of parahippocampal | IVW | -5.3315(-9.2083, -1.4547) | 1.97796 | 0.00703 |
| Surface area of parsopercularis | IVW | -4.9521(-18.4385, 8.5344) | 6.88083 | 0.47172 |
| Surface area of parsorbitalis | IVW | 0.0757(-3.0343, 3.1857) | 1.58673 | 0.96194 |
| Surface area of parstriangularis | IVW | 4.7812(-3.4357, 12.9982) | 4.19232 | 0.25409 |
| Surface area of pericalcarine | IVW | 3.0104(-16.0302, 22.0509) | 9.71459 | 0.75665 |
| Surface area of postcentral | IVW | 1.6551(-15.7977, 19.108) | 8.90451 | 0.85254 |
| Surface area of posteriorcingulate | IVW | 0.5853(-5.1764, 6.3471) | 2.93969 | 0.84217 |
| Surface area of precentral | IVW | 1.6336(-15.1851, 18.4522) | 8.58094 | 0.84902 |
| Surface area of precuneus | IVW | -3.393(-17.7637, 10.9777) | 7.33197 | 0.64353 |
| Surface area of rostralanteriorcingulate | IVW | -0.5463(-5.4222, 4.3296) | 2.48771 | 0.82619 |
| Surface area of rostralmiddlefrontal | IVW | -10.297(-42.1047, 21.5108) | 16.22844 | 0.52575 |
| Surface area of superiorfrontal | IVW | 1.2168(-19.0072, 21.4407) | 10.31833 | 0.90613 |
| Surface area of superiorparietal | IVW | -17.0388(-37.5791, 3.5015) | 10.47975 | 0.10398 |
| Surface area of superiortemporal | IVW | 5.7342(-6.6747, 18.1431) | 6.33106 | 0.36508 |
| Surface area of supramarginal | IVW | -18.1344(-55.3941, 19.1252) | 19.01001 | 0.34011 |
| Surface area of temporalpole | IVW | -0.9155(-5.2512, 3.4201) | 2.21207 | 0.67897 |
| Surface area of transversetemporal | IVW | 1.0955(-1.5378, 3.7287) | 1.34349 | 0.41484 |
| Thickness of bankssts | IVW | 0.0009(-0.0052, 0.007) | 0.00312 | 0.76707 |
| Thickness of caudalanteriorcingulate | IVW | 0.0045(-0.0115, 0.0205) | 0.00816 | 0.58383 |
| Thickness of caudalmiddlefrontal | IVW | -0.0041(-0.009, 0.0008) | 0.00249 | 0.10017 |
| Thickness of cuneus | IVW | -0.0075(-0.013, -0.002) | 0.0028 | 0.00719 |
| Thickness of entorhinal | IVW | 0.0251(0.0093, 0.041) | 0.0081 | 0.00191 |
| Thickness of frontalpole | IVW | -0.0056(-0.0165, 0.0054) | 0.00558 | 0.31681 |
| Thickness of fusiform | IVW | 0.0003(-0.005, 0.0057) | 0.00273 | 0.90877 |
| Thickness of inferiorparietal | IVW | 0.0001(-0.0088, 0.0089) | 0.00452 | 0.99028 |
| Thickness of inferiortemporal | IVW | 0.0043(-0.0025, 0.0111) | 0.00348 | 0.21715 |
| Thickness of insula | IVW | 0.0033(-0.0044, 0.011) | 0.00392 | 0.39671 |
| Thickness of isthmuscingulate | IVW | -0.0003(-0.0091, 0.0084) | 0.00446 | 0.94127 |
| Thickness of lateraloccipital | IVW | -0.0005(-0.0072, 0.0063) | 0.00344 | 0.88553 |
| Thickness of lateralorbitofrontal | IVW | 0.0062(0.0003, 0.0121) | 0.00303 | 0.04105 |
| Thickness of lingual | IVW | -0.0063(-0.0112, -0.0015) | 0.00249 | 0.01093 |
| Thickness of medialorbitofrontal | IVW | -0.0019(-0.0096, 0.0058) | 0.00394 | 0.62801 |
| Thickness of middletemporal | IVW | -0.0008(-0.0062, 0.0047) | 0.00278 | 0.77631 |
| Thickness of paracentral | IVW | -0.0005(-0.006, 0.005) | 0.0028 | 0.86328 |
| Thickness of parahippocampal | IVW | 0.0047(-0.009, 0.0185) | 0.00701 | 0.50137 |
| Thickness of parsopercularis | IVW | -0.0072(-0.0117, -0.0027) | 0.0023 | 0.00171 |
| Thickness of parsorbitalis | IVW | -0.0045(-0.0121, 0.0032) | 0.00389 | 0.25191 |
| Thickness of parstriangularis | IVW | -0.0058(-0.0109, -0.0008) | 0.00255 | 0.02205 |
| Thickness of pericalcarine | IVW | -0.0086(-0.0141, -0.0031) | 0.0028 | 0.00208 |
| Thickness of postcentral | IVW | -0.0011(-0.0055, 0.0033) | 0.00224 | 0.6233 |
| Thickness of posteriorcingulate | IVW | 0.0037(-0.0023, 0.0098) | 0.0031 | 0.22604 |
| Thickness of precentral | IVW | 0.0016(-0.0034, 0.0065) | 0.00251 | 0.53247 |
| Thickness of precuneus | IVW | 0.0013(-0.003, 0.0056) | 0.00219 | 0.55078 |
| Thickness of rostralanteriorcingulate | IVW | 0.007(-0.0023, 0.0163) | 0.00475 | 0.14083 |
| Thickness of rostralmiddlefrontal | IVW | -0.0025(-0.0068, 0.0018) | 0.00219 | 0.25904 |
| Thickness of superiorfrontal | IVW | -0.0003(-0.0048, 0.0041) | 0.00226 | 0.88287 |
| Thickness of superiorparietal | IVW | -0.002(-0.0059, 0.002) | 0.00201 | 0.32737 |
| Thickness of superiortemporal | IVW | 0.0012(-0.0042, 0.0067) | 0.00278 | 0.66173 |
| Thickness of supramarginal | IVW | 0.0028(-0.0053, 0.011) | 0.00414 | 0.49321 |
| Thickness of temporalpole | IVW | 0.0143(0.0005, 0.0281) | 0.00703 | 0.04204 |
| Thickness of transversetemporal | IVW | -0.002(-0.0109, 0.0068) | 0.0045 | 0.64971 |
| **PLF** |  |  |  |  |
| Surface area of bankssts | IVW | -4.6527(-10.8684, 1.5631) | 3.17132 | 0.14235 |
| Surface area of caudalanteriorcingulate | IVW | 0.8568(-4.1554, 5.8689) | 2.55723 | 0.73759 |
| Surface area of caudalmiddlefrontal | IVW | 3.4191(-9.7865, 16.6247) | 6.73755 | 0.61183 |
| Surface area of cuneus | IVW | 6.3189(-2.9481, 15.5859) | 4.72808 | 0.1814 |
| Surface area of entorhinal | IVW | -3.5438(-9.024, 1.9364) | 2.79601 | 0.20499 |
| Surface area of frontalpole | IVW | 0.046(-1.6728, 1.7648) | 0.87693 | 0.95817 |
| Surface area of fusiform | IVW | 2.2345(-10.3295, 14.7985) | 6.41019 | 0.7274 |
| Surface area of inferiorparietal | IVW | 3.1376(-18.3159, 24.591) | 10.94565 | 0.77438 |
| Surface area of inferiortemporal | IVW | 8.0101(-13.2647, 29.2848) | 10.85446 | 0.46054 |
| Surface area of insula | IVW | 4.4752(-4.0752, 13.0257) | 4.36248 | 0.30496 |
| Surface area of isthmuscingulate | IVW | -2.0476(-7.8069, 3.7118) | 2.93845 | 0.48592 |
| Surface area of lateraloccipital | IVW | 12.4366(-13.9485, 38.8216) | 13.46177 | 0.35557 |
| Surface area of lateralorbitofrontal | IVW | 1.8908(-7.3306, 11.1122) | 4.70479 | 0.68777 |
| Surface area of lingual | IVW | -1.8644(-17.1928, 13.464) | 7.82062 | 0.81157 |
| Surface area of medialorbitofrontal | IVW | -0.7185(-7.569, 6.1321) | 3.49516 | 0.83714 |
| Surface area of middletemporal | IVW | 0.4444(-12.2585, 13.1472) | 6.48107 | 0.94534 |
| Surface area of paracentral | IVW | 0.0239(-7.8052, 7.853) | 3.99445 | 0.99523 |
| Surface area of parahippocampal | IVW | -6.0644(-9.9393, -2.1895) | 1.97698 | 0.00216 |
| Surface area of parsopercularis | IVW | -5.2686(-15.2456, 4.7084) | 5.09031 | 0.30066 |
| Surface area of parsorbitalis | IVW | 0.2583(-3.088, 3.6046) | 1.7073 | 0.87976 |
| Surface area of parstriangularis | IVW | 5.5423(-2.6471, 13.7318) | 4.17831 | 0.18469 |
| Surface area of pericalcarine | IVW | 3.799(-11.08, 18.678) | 7.59134 | 0.61677 |
| Surface area of postcentral | IVW | 0.7876(-13.5052, 15.0804) | 7.29225 | 0.91399 |
| Surface area of posteriorcingulate | IVW | 2.769(-2.9798, 8.5179) | 2.9331 | 0.34514 |
| Surface area of precentral | IVW | -0.8009(-17.5814, 15.9795) | 8.56145 | 0.92547 |
| Surface area of precuneus | IVW | -1.8024(-16.1492, 12.5444) | 7.31981 | 0.8055 |
| Surface area of rostralanteriorcingulate | IVW | -0.5775(-5.4402, 4.2852) | 2.48099 | 0.81594 |
| Surface area of rostralmiddlefrontal | IVW | -12.2811(-37.724, 13.1619) | 12.9811 | 0.34411 |
| Surface area of superiorfrontal | IVW | -4.8167(-24.9956, 15.3623) | 10.2954 | 0.63989 |
| Surface area of superiorparietal | IVW | -10.6043(-31.1038, 9.8952) | 10.45892 | 0.31063 |
| Surface area of superiortemporal | IVW | 7.8992(-6.2545, 22.053) | 7.22129 | 0.27401 |
| Surface area of supramarginal | IVW | -7.5676(-27.9153, 12.78) | 10.38146 | 0.46603 |
| Surface area of temporalpole | IVW | -1.6782(-4.4783, 1.1219) | 1.42861 | 0.2401 |
| Surface area of transversetemporal | IVW | 0.7672(-1.8374, 3.3718) | 1.32889 | 0.56372 |
| Thickness of bankssts | IVW | 0.0005(-0.0056, 0.0067) | 0.00315 | 0.8626 |
| Thickness of caudalanteriorcingulate | IVW | 0.0061(-0.0065, 0.0187) | 0.0064 | 0.34083 |
| Thickness of caudalmiddlefrontal | IVW | -0.0029(-0.0078, 0.002) | 0.0025 | 0.24213 |
| Thickness of cuneus | IVW | -0.0077(-0.0132, -0.0021) | 0.00282 | 0.00659 |
| Thickness of entorhinal | IVW | 0.0246(0.0027, 0.0465) | 0.01115 | 0.0274 |
| Thickness of frontalpole | IVW | -0.0055(-0.0174, 0.0065) | 0.00608 | 0.36892 |
| Thickness of fusiform | IVW | 0.0023(-0.003, 0.0076) | 0.00272 | 0.4016 |
| Thickness of inferiorparietal | IVW | 0.002(-0.0039, 0.0079) | 0.003 | 0.51031 |
| Thickness of inferiortemporal | IVW | 0.0055(-0.0004, 0.0114) | 0.00302 | 0.06808 |
| Thickness of insula | IVW | 0.0029(-0.0039, 0.0097) | 0.00347 | 0.39841 |
| Thickness of isthmuscingulate | IVW | -0.0073(-0.0171, 0.0024) | 0.00499 | 0.14096 |
| Thickness of lateraloccipital | IVW | 0.0005(-0.004, 0.005) | 0.00229 | 0.81891 |
| Thickness of lateralorbitofrontal | IVW | 0.0055(-0.0004, 0.0114) | 0.00302 | 0.0672 |
| Thickness of lingual | IVW | -0.0063(-0.0119, -0.0006) | 0.0029 | 0.03117 |
| Thickness of medialorbitofrontal | IVW | -0.0018(-0.0086, 0.0049) | 0.00344 | 0.59204 |
| Thickness of middletemporal | IVW | -0.0009(-0.0064, 0.0045) | 0.0028 | 0.73502 |
| Thickness of paracentral | IVW | -0.0019(-0.0074, 0.0037) | 0.00283 | 0.50775 |
| Thickness of parahippocampal | IVW | 0.0103(-0.0061, 0.0267) | 0.00837 | 0.21826 |
| Thickness of parsopercularis | IVW | -0.0077(-0.0123, -0.0031) | 0.00235 | 0.00105 |
| Thickness of parsorbitalis | IVW | -0.0038(-0.0132, 0.0057) | 0.00484 | 0.43781 |
| Thickness of parstriangularis | IVW | -0.0045(-0.0096, 0.0006) | 0.00259 | 0.08193 |
| Thickness of pericalcarine | IVW | -0.0068(-0.0138, 0.0003) | 0.0036 | 0.06009 |
| Thickness of postcentral | IVW | -0.0012(-0.0057, 0.0032) | 0.00227 | 0.58696 |
| Thickness of posteriorcingulate | IVW | 0.0003(-0.0064, 0.0069) | 0.00338 | 0.94037 |
| Thickness of precentral | IVW | 0.0015(-0.0034, 0.0064) | 0.00251 | 0.55656 |
| Thickness of precuneus | IVW | -0.0004(-0.0046, 0.0038) | 0.00212 | 0.85393 |
| Thickness of rostralanteriorcingulate | IVW | 0.0077(-0.0016, 0.0169) | 0.0047 | 0.10333 |
| Thickness of rostralmiddlefrontal | IVW | -0.0013(-0.0056, 0.003) | 0.00219 | 0.55296 |
| Thickness of superiorfrontal | IVW | -0.0003(-0.0056, 0.005) | 0.00271 | 0.90191 |
| Thickness of superiorparietal | IVW | -0.0027(-0.0066, 0.0011) | 0.00199 | 0.16706 |
| Thickness of superiortemporal | IVW | 0.0005(-0.005, 0.006) | 0.00281 | 0.84735 |
| Thickness of supramarginal | IVW | 0.003(-0.0025, 0.0084) | 0.00278 | 0.28708 |
| Thickness of temporalpole | IVW | 0.0163(-0.003, 0.0355) | 0.00982 | 0.09807 |
| Thickness of transversetemporal | IVW | -0.0017(-0.0141, 0.0107) | 0.00633 | 0.78622 |

**ALT**, alanine transaminase; **IVW**, inverse-variance weighted; **NAFLD**, non-alcoholic fatty liver disease; **PLF**, percent liver fat; **SE**, Standard error.
