# Supplementary material for: Causal effects of nonalcoholic fatty liver disease on cerebral cortical structure: a Mendelian randomization analysis
Source: Front Endocrinol (Lausanne). 2023 Nov 1;14:1276576. doi: 10.3389/fendo.2023.1276576 (PMC10646496; doi:10.3389/fendo.2023.1276576)
Supplement: Supplementary Table 4 — Inverse-variance weighted, MR-Egger and weighted median estimates of the significant and nominally significant Mendelian randomization estimates. [file Table_4.docx]

**Table S4.** Inverse-variance weighted, MR-Egger and weighted median estimates of the significant and nominally significant Mendelian randomization estimates

| **Exposures**  **Outcomes** | **Method** | **β (95%CI)** | **SE** | **P value** |
| --- | --- | --- | --- | --- |
| **ALT** |  |  |  |  |
| Surface area of parahippocampal gyrus | IVW | -60.4594(-104.9948--15.9239) | 22.72216 | 0.0078 |
| Surface area of parahippocampal gyrus | MR Egger | -91.2453(-221.7929-39.3023) | 66.60591 | 0.22902 |
| Surface area of parahippocampal gyrus | Weighted median | -66.5489(-122.575--10.5228) | 28.58474 | 0.01991 |
| Thickness of pars opercularis | IVW | -0.0861(-0.1402--0.0319) | 0.02764 | 0.00185 |
| Thickness of pars opercularis | MR Egger | -0.1482(-0.3081-0.0117) | 0.0816 | 0.12902 |
| Thickness of pars opercularis | Weighted median | -0.0859(-0.1525--0.0193) | 0.03396 | 0.01143 |
| Thickness of pars orbitalis | IVW | -0.1023(-0.1874--0.0172) | 0.04343 | 0.01849 |
| Thickness of pars orbitalis | MR Egger | -0.0513(-0.3039-0.2012) | 0.12886 | 0.70691 |
| Thickness of pars orbitalis | Weighted median | -0.1015(-0.2052-0.0023) | 0.05294 | 0.0553 |
| Thickness of pericalcarine cortex | IVW | -0.0913(-0.1767--0.006) | 0.04356 | 0.03603 |
| Thickness of pericalcarine cortex | MR Egger | -0.3288(-0.5101--0.1474) | 0.09253 | 0.01633 |
| Thickness of pericalcarine cortex | Weighted median | -0.1423(-0.2249--0.0598) | 0.04213 | 0.00073 |
| **NAFLD** |  |  |  |  |
| Surface area of parahippocampal gyrus | IVW | -5.3315(-9.2083--1.4547) | 1.97796 | 0.00703 |
| Surface area of parahippocampal gyrus | MR Egger | -1.3309(-14.6266-11.9647) | 6.7835 | 0.87666 |
| Surface area of parahippocampal gyrus | Weighted median | -5.2041(-9.2643--1.1438) | 2.07154 | 0.012 |
| Thickness of cuneus | IVW | -0.0075(-0.013--0.002) | 0.0028 | 0.00719 |
| Thickness of cuneus | MR Egger | 0.0047(-0.0145-0.0239) | 0.0098 | 0.71719 |
| Thickness of cuneus | Weighted median | -0.0065(-0.0122--0.0007) | 0.00294 | 0.02769 |
| Thickness of entorhinal cortex | IVW | 0.0251(0.0093-0.041) | 0.0081 | 0.00191 |
| Thickness of entorhinal cortex | MR Egger | 0.0437(-0.0111-0.0985) | 0.02796 | 0.36266 |
| Thickness of entorhinal cortex | Weighted median | 0.0269(0.0098-0.044) | 0.00871 | 0.00203 |
| Thickness of lateral orbitofrontal cortex | IVW | 0.0062(0.0003-0.0121) | 0.00303 | 0.04105 |
| Thickness of lateral orbitofrontal cortex | MR Egger | 0.0032(-0.0166-0.023) | 0.0101 | 0.80726 |
| Thickness of lateral orbitofrontal cortex | Weighted median | 0.0059(-0.0004-0.0122) | 0.00321 | 0.06795 |
| Thickness of lingual gyrus | IVW | -0.0063(-0.0112--0.0015) | 0.00249 | 0.01093 |
| Thickness of lingual gyrus | MR Egger | 0.0015(-0.0149-0.0179) | 0.00837 | 0.88373 |
| Thickness of lingual gyrus | Weighted median | -0.0056(-0.0109--0.0004) | 0.00268 | 0.03594 |
| Thickness of pars opercularis | IVW | -0.0072(-0.0117--0.0027) | 0.0023 | 0.00171 |
| Thickness of pars opercularis | MR Egger | -0.0046(-0.0205-0.0113) | 0.0081 | 0.67319 |
| Thickness of pars opercularis | Weighted median | -0.0071(-0.0119--0.0023) | 0.00244 | 0.00346 |
| Thickness of par striangularis | IVW | -0.0058(-0.0109--0.0008) | 0.00255 | 0.02205 |
| Thickness of par striangularis | MR Egger | -0.0024(-0.02-0.0151) | 0.00895 | 0.83154 |
| Thickness of par striangularis | Weighted median | -0.0058(-0.0113--0.0003) | 0.00278 | 0.03721 |
| Thickness of pericalcarine cortex | IVW | -0.0086(-0.0141--0.0031) | 0.0028 | 0.00208 |
| Thickness of pericalcarine cortex | MR Egger | -0.0102(-0.0294-0.009) | 0.0098 | 0.48844 |
| Thickness of pericalcarine cortex | Weighted median | -0.0087(-0.0147--0.0027) | 0.00304 | 0.00426 |
| Thickness of temporal pole | IVW | 0.0143(0.0005-0.0281) | 0.00703 | 0.04204 |
| Thickness of temporal pole | MR Egger | 0.0137(-0.0333-0.0607) | 0.024 | 0.66978 |
| Thickness of temporal pole | Weighted median | 0.0168(0.0019-0.0318) | 0.00763 | 0.02737 |
| **PLF** |  |  |  |  |
| Ssurface area of full cortex | IVW | -900.7396(-1625.1751--176.3041) | 369.61 | 0.01481 |
| Surface area of full cortex | MR Egger | -1378.6372(-2520.1843--237.0902) | 582.422 | 0.04982 |
| Surface area of full cortex | Weighted median | -805.624(-1702.873-91.625) | 457.7801 | 0.07843 |
| Surface area of parahippocampal gyrus | IVW | -6.0644(-9.9393--2.1895) | 1.97698 | 0.00216 |
| Surface area of parahippocampal gyrus | MR Egger | -3.4746(-9.5875-2.6383) | 3.11883 | 0.30204 |
| Surface area of parahippocampal gyrus | Weighted median | -4.8754(-9.4719--0.2789) | 2.34515 | 0.03763 |
| Thickness of cuneus | IVW | -0.0077(-0.0132--0.0021) | 0.00282 | 0.00659 |
| Thickness of cuneus | MR Egger | -0.0089(-0.0176--0.0002) | 0.00444 | 0.08486 |
| Thickness of cuneus | Weighted median | -0.0074(-0.0142--0.0007) | 0.00344 | 0.03118 |
| Thickness of entorhinal cortex | IVW | 0.0246(0.0027-0.0465) | 0.01115 | 0.0274 |
| Thickness of entorhinal cortex | MR Egger | 0.023(-0.0138-0.0597) | 0.01877 | 0.26066 |
| Thickness of entorhinal cortex | Weighted median | 0.0282(0.0086-0.0478) | 0.00999 | 0.00476 |
| Thickness of lingual gyrus | IVW | -0.0063(-0.0119--0.0006) | 0.0029 | 0.03117 |
| Thickness of lingual gyrus | MR Egger | -0.0066(-0.0162-0.003) | 0.00488 | 0.21921 |
| Thickness of lingual gyrus | Weighted median | -0.0061(-0.0122-0) | 0.00312 | 0.05096 |
| Thickness of pars opercularis | IVW | -0.0077(-0.0123--0.0031) | 0.00235 | 0.00105 |
| Thickness of pars opercularis | MR Egger | -0.0082(-0.0155--0.0009) | 0.00373 | 0.06344 |
| Thickness of pars opercularis | Weighted median | -0.008(-0.0136--0.0025) | 0.00284 | 0.0046 |
