# Supplementary material for: Causal effects of nonalcoholic fatty liver disease on cerebral cortical structure: a Mendelian randomization analysis
Source: Front Endocrinol (Lausanne). 2023 Nov 1;14:1276576. doi: 10.3389/fendo.2023.1276576 (PMC10646496; doi:10.3389/fendo.2023.1276576)
Supplement: Supplementary Table 5 — MR-PRESSO estimates of the significant and nominally significant Mendelian randomization estimates. [file Table_5.docx]

**Table S5.** MR-PRESSO estimates of the significant and nominally significant Mendelian randomization estimates

| **Outcomes** | **RSSobs** | **MR-PRESSO global test p value** |
| --- | --- | --- |
| Surface area of full cortex | 17.46414 | 0.67 |
| Surface area of parahippocampal gyrus | 11.35355 | 0.94 |
| Thickness of cuneus | 22.12922 | 0.38 |
| Thickness of entorhinal cortex | 27.51139 | 0.15 |
| Thickness of lateral orbitofrontal cortex | 10.84953 | 0.95 |
| Thickness of lingual gyrus | 19.83627 | 0.5 |
| Thickness of pars opercularis | 18.9929 | 0.57 |
| Thickness of pars orbitalis | 23.12393 | 0.31 |
| Thickness of pars triangularis | 8.716449 | 0.99 |
| Thickness of pericalcarine cortex | 32.67379 | 0.05 |
| Thickness of temporal pole | 29.31889 | 0.11 |
